# Exploring EEG and ECG in Music Listening: A Scoping Review

**DOI:** 10.1111/psyp.70385

**Published:** 2026-09-03

**Authors:** Scott R. Leimroth, Robert J. Barry, Frances M. De Blasio, Timothy P. Byron

**Affiliations:** ^1^ Brain & Behaviour Research Institute and School of Psychology, University of Wollongong Wollongong New South Wales Australia

**Keywords:** ECG, EEG, guidelines, methodology, music listening, scoping review

## Abstract

Music listening, a common daily activity recognized for its emotional impact and ability to evoke memories, engages physiological and psychological processes. Concurrent electroencephalography (EEG) and electrocardiography (ECG) during music listening can inform our understanding of these processes. However, the insights offered by such simultaneous recordings, and the extent to which existing studies align with published methodological guidelines, remain unclear. To address this gap, we conducted a scoping review of peer‐reviewed research examining physiological responses to passive music listening using combined EEG and ECG measures in healthy adult non‐musicians. A structured search of five databases identified 18 papers. Data on methods, participant demographics, musical stimuli, and EEG/ECG measures were extracted and compared against established guidelines to clarify the studies' methodological rigor. EEG findings were highly heterogeneous, spanning spectral power, lateralized effects, and event‐related potentials, with limited overlap in analytical focus. Cardiac outcomes were similarly mixed, with studies reporting increases, decreases, or no change in heart rate depending on stimulus and context. Only a small subset of studies examined brain‐heart coupling directly, with preliminary evidence of synchronization between neural and cardiac rhythms during music listening, leaving the central question of how brain and heart respond together to music largely open. Our review identified significant methodological inconsistencies and a lack of integration between the two measures, highlighting the challenges of this interdisciplinary research. Advancing research in both music and psychophysiology requires improving alignment between methodologies from these distinct areas by following established standards. These findings highlight a critical need for greater methodological standardization to build a more reliable and replicable understanding of music's psychophysiological effects.

## Impact Statement

This scoping review is the first to map concurrent EEG and ECG during music listening in healthy adults. Comparing 18 studies against published guidelines, we expose methodological inconsistencies and identify emerging evidence of brain and heart coupling. The reporting standards we propose strengthen EEG, ECG, and music‐as‐stimulus research individually and together.

## 1 Introduction

Music's complex structure and profound impact on the human mind and body make it a uniquely rich stimulus for scientific experiments exploring cognitive processes, emotional states, and physiological responses. Electroencephalography (EEG) and electrocardiography (ECG), when applied with methodological rigor, offer important insight into the temporal dynamics of brain activity and cardiac responses during musical engagement, improving our understanding of the interaction between cognitive processes, emotional states, and physiological responses.

People listen to music to manage their mood, gain self‐awareness, and connect with others, highlighting its significant psychological functions (Schäfer et al. 2013). The music‐listening experience ranges from incidental to attentive, activating brain regions associated with auditory processing, working memory, and emotional processing, and evoking cognitive, emotional, and physiological responses (Hallam 2019). Responses to a piece of music can vary between listeners and occasions, depending on context and interpretation, influenced by different elements of the music. Prince (1972) identified nine interacting variables influencing response to music: musical aptitude, musical memory, personality, attitude, expectation, state of attention, affective responses, associative responses, and perceptual and learning processes. Complementing this perspective, the BRECVEMAC framework (Juslin 2025) offers a theoretical model explaining how music influences emotions through nine psychological mechanisms: brain stem reflex, rhythmic entrainment, evaluative conditioning, emotional contagion, visual imagery, episodic memory, musical expectancy, aesthetic judgment, and cognitive goal appraisal. These mechanisms account for instinctual physiological responses to sound, the influence of rhythm and tempo, associations with past experiences, and emotional contagion which occurs when we mirror the emotions we perceive in the music. Additionally, it considers how music evokes mental imagery, triggers specific memories, engages our expectations, elicits aesthetic appreciation, and affects a listener's personal goals. These frameworks highlight the multifaceted nature of the factors contributing to the wide variability in responses to music. Given the complexity and variability inherent in musical stimuli and individual responses, it is important that researchers adopt a systematic and rigorous approach when investigating the interplay between music and physiological responses. Following established guidelines ensures that research findings are reliable and that the intricate relationship between music and physiological responses is accurately captured.

### 1.1 Music, EEG, and ECG

EEG techniques have been instrumental in investigating brain responses to musical stimuli, providing insights into various aspects of sound processing and cognition. However, these insights are only robust when researchers follow established methodological guidelines, ensuring that findings are both sound and replicable. EEG responses to music have been found to reflect cognitive processes associated with emotional processing (Tsang et al. 2001), expectation (Rohrmeier and Koelsch 2012), attention (Jäncke et al. 2018), the recognition of structure within musical patterns (Zatorre and Salimpoor 2013), and musical experience (Pallesen et al. 2015), among others. EEG measures are well‐suited for studying music listening due to their high temporal resolution, capturing brain activity changes in milliseconds, enabling real‐time tracking of neural responses to the inherently temporal nature of music. In a review of EEG studies of musical perception, González et al. (2021) found that measures of spectral power across various cortical areas suggest that musical processing involves both local and distant neural networks. These networks communicate through different EEG frequency bands, such as alpha power changes in parieto/occipital and fronto/temporal regions, beta power changes in the right parietal/temporal cortex, and gamma power changes in the right parietal region. The type of tonal music being heard influences these spectral power alterations across specific cortical areas bilaterally. Also, in the context of auditory stimuli, particularly music listening and recognition, a review by Freitas et al. (2018) summarized several key event‐related potentials (ERPs) that had been identified in the literature. The N1 wave, occurring 80–110 ms after stimulus onset and originating in the auditory cortex, detects sound features and changes. The P2 wave, appearing 160–200 ms post‐stimulus, plays a role in stimulus evaluation and cognitive processes like working memory. The P3 wave, including components like P3a and P3b, emerges 300–400 ms after stimulus onset and is associated with selective attention, recognition, and memory processes. The N400 wave, occurring 200–600 ms post‐stimulus, is involved in processing meaning across various cognitive levels (Freitas et al. 2018).

A substantial body of research confirms the relationship between musical auditory stimulation and cardiac response (Hodges 2010; Koelsch and Jäncke 2015; Mojtabavi et al. 2020). Changes in cardiac activity related to the parasympathetic nervous system response to incoming sound information are consistently found (da Silva et al. 2014; Koelsch and Jäncke 2015; Krabs et al. 2015; Zhao and Kuhl 2020). However, physiological responses to music are not consistent (Hodges 2010; Mojtabavi et al. 2020). Different types of music have varying effects on the sympathetic nervous system (SNS) and parasympathetic nervous system (PNS), depending on factors such as tempo, mode, and emotional valence (Mojtabavi et al. 2020; Wang and Huang 2014). This inconsistency of response is commonly attributed to individual differences in autonomic nervous system (ANS) response (Lacey and Lacey 1978), but is also likely influenced by the subjective interpretation of the music (Meyer 1956), a factor often overlooked. Subjective interpretation and listener responses to music can be influenced by methodological inconsistencies and variations in aspects of the music stimulus (Hofbauer and Rodriguez 2023; Kellaris and Kent 1993). Beyond individual and interpretive factors, the temporal structure and rhythmic patterns of music influence physiological, cognitive, and emotional responses, making music a compelling stimulus for investigating coordinated neural and cardiac dynamics.

### 1.2 Rhythmic Patterns in EEG, ECG, and Music

Rhythmic patterns are major determinants of physiological changes to music (Bernardi et al. 2009; Etzel et al. 2006; Gomez and Danuser 2007; Juslin et al. 2022). Music's coherence relies on its temporal flow, rendering its structures dynamic rather than static or reversible (Laske and Drummond 1980). Music, EEG, and ECG share a temporal nature, involving phenomena unfolding over time and characterized by various frequencies and rhythmic patterns. With high temporal resolution, EEG effectively captures the brain's rapid responses to music's temporal dynamics. ECG complements EEG by providing insights into the heart's response to music, reflecting the autonomic nervous system's regulation through measures such as heart rate (HR), blood pressure (BP), and heart rate variability (HRV). Entrainment refers to the synchronization of an organism's internal rhythms with external stimuli. In the context of music, rhythmic entrainment is the alignment of neural and physiological rhythms to the beat of the music, which can enhance motor coordination, emotional regulation, and cognitive processing (Thaut 2015). This synchronization can be observed in both EEG and ECG data, where neural and cardiac oscillations can align with the tempo and rhythm of the music, highlighting the interconnectedness of brain and cardiac responses to musical stimuli (Bernardi et al. 2006; Nozaradan et al. 2012). Numerous studies have investigated the effects of cardiac activity on EEG and vice versa (Critchley and Harrison 2013; Park et al. 2014; Schandry and Montoya 1996), however, research on their interaction during music listening remains limited.

### 1.3 Interaction Between Heart and Brain

The “baroreceptor hypothesis” proposed that heart activity, specifically baroreceptor inputs (pressure‐sensitive receptors in the aortic arch and carotid sinus that fire in synchrony with the cardiac cycle), can influence cognitive functions like attention and memory (Garfinkel et al. 2013; Lacey and Lacey 1970; Li et al. 2018; Suarez‐Roca et al. 2021; Wölk and Velden 1987). Research indicates that stimuli presented at different phases of the cardiac cycle affect cognitive performance, suggesting a synchronization between alpha brain waves and baroreceptor activity that may impact sensory information transmission (Lacey and Lacey 1978, 2017; Wölk and Velden 1987). The phase of the cardiac cycle influences cognitive control mechanisms, affecting processing speed and perceptual processes such as auditory threshold and temporal judgment (Al et al. 2021; Sadeghi et al. 2023; Sandman 1984; Saxon 1970). Cardiac afferent signaling influences cognitive processes including attention, arousal, motivation, emotions, bodily self‐awareness, self‐related cognition, somatosensory processing, and perception (Al et al. 2021; Azzalini et al. 2019; Critchley and Garfinkel 2018; Kunzendorf et al. 2019; Schandry and Montoya 1996).

The interaction between the heart and brain is bidirectional, encompassing not only the effect of the heart on the brain, through mechanisms like the baroreceptor hypothesis, but also the influence of the brain on the heart, primarily mediated by the vagus nerve. Lacey and Lacey (2017) suggested that attention induces cardiac deceleration, while cognitive work produces acceleration. Patron et al. (2019) found that delta oscillations in the frontal cortex influence heart rate deceleration at rest, suggesting increased cardiac vagal control and a regulatory link between brain activity and cardiac function.

With evidence suggesting that communication between the heart and brain shapes cognitive experience and emotional response, music serves as an ideal medium for this exploration due to its emotional impact and ability to engage complex cognitive processes. This scoping review examines studies assessing both EEG and ECG measures to map the current literature and uncover the links between brain‐heart interaction during music listening.

Combining EEG and ECG concurrently allows investigators to assess two complementary views of the listener's response to music, and it is worth stating what each measure contributes, and why recording them together is more informative than either alone. EEG records the brain's response and processing of the musical stimulus with high temporal resolution, capturing cortical activity as a piece unfolds, while ECG, and the heart‐rate and heart‐rate‐variability measures derived from this, reflect the listener's autonomic state and the balance of sympathetic and parasympathetic drive that accompanies changes in arousal and emotion. Recorded on their own, each signal is hard to interpret. For example, a change in cortical alpha might reflect stimulus processing or a general shift in arousal, and a change in heart rate could reflect the emotional impact of the music or an incidental change in respiration or posture. When recorded together, EEG and ECG can be cross‐referenced to investigate whether neural and autonomic responses to the same musical event covary, lag one another, or become entrained to a shared rhythm. Beyond the music literature, the concurrent recording of cortical and cardiac activity has already shown that the brain produces a small response to each heartbeat, and that this response relates to perception, emotion, and self‐related processing (Azzalini et al. 2019; Critchley and Harrison 2013; Park et al. 2014). It is this covariation, rather than either signal individually, that best reflects the bidirectional brain‐heart communication outlined above, that motivates our focus on studies that have recorded both measures concurrently.

### 1.4 Objective

This scoping review clarifies how the interactions between music listening, physiology, and cognition have been conceptualized and investigated. It systematically maps the existing peer‐reviewed literature on the simultaneous use of EEG and ECG in measuring physiological, cognitive, and emotional response during music listening in healthy adults. It evaluates the methodologies employed, in relation to published guidelines, to identify gaps, improve research practices, and guide future studies towards a better understanding of the complex interactions between music, physiology, and cognition. This review explores specific questions, including the types of studies conducted, the populations examined, the methodologies used for EEG and ECG measurement, the details of the stimulus and study design, and the interaction between EEG and ECG measures. Consistent with the points made above, particular attention is given to whether, and how, studies analysed the two measures in relation to one another rather than reporting them separately. The goal is to highlight areas needing further exploration and to improve the methodological rigor of future studies by identifying where current practice falls short of published standards.

## 2 Methods

This scoping review followed the methodology outlined by Peters et al. (2022) and adhered to the guidelines for evidence synthesis provided in the Joanna Briggs Institute (JBI) manual (Aromataris et al. 2024). The Population, Concept, and Context (PCC) framework was adopted to set the eligibility criteria, title, and research questions for the study (see Supporting Information—Appendix S1). This approach was guided by the established scoping review framework (Arksey and O'Malley 2005; Levac et al. 2010) and the Preferred Reporting Items for Systematic Reviews and Meta‐Analyses (PRISMA) extension for Scoping Reviews (PRISMA‐ScR) (Tricco et al. 2018).

### 2.1 Protocol and Registration

The study protocol was determined a priori and was preregistered online at https://osf.io/yx3rh (associated project https://osf.io/xtznm) and followed the OSF preregistration template (Bowman et al. 2020). The protocol followed the standards set in the PRISMA‐ScR (Tricco et al. 2018).

### 2.2 Eligibility Criteria

Eligible articles were required to meet the following criteria:

1. Published studies in English in a peer‐reviewed journal. We excluded replications, reviews, book chapters, conference abstracts, dissertation theses, pre‐prints, and studies that were protocols or methodological only.
2. Both EEG and ECG data were recorded concurrently during the same experimental condition or event. The relationship between these two measures is fundamental for the scope of this review, reflecting our question regarding the extent of the interaction between the two measures. It should be noted that this criterion retains studies in which the ECG signal was used solely as an internal timing marker for cardiac‐locked EEG analysis, rather than as a cardiac outcome measure. These studies were retained because they met the concurrent recording criterion and excluding them would have been inconsistent with the pre‐registered eligibility framework, although they do not contribute cardiac outcome data to the synthesis.
3. Conscious and awake, healthy adult (18+) human non‐musician participants. These criteria were selected to exclude animal studies and machine learning or other models. A healthy population was specified to avoid confounds associated with clinical populations. Adults were defined as those over 18 years of age and were specified to avoid confounds associated with significant physical, psychological, and emotional changes during childhood and adolescent development. Musicians were excluded to avoid confounds associated with enhanced auditory processing skills, heightened sensitivity to musical nuances, and potential training‐induced neuroplastic changes in brain structure and function (Besson et al. 2007; Grassi et al. 2025; Herholz and Zatorre 2012; Kraus and Chandrasekaran 2010). Studies that referred to participants as musicians (Zhang et al. 2020) or having musical training, or that specifically selected for participants with musical training or abilities, were excluded. Musical training is one of the most reliable moderators of neural, and to a lesser extent autonomic, responses to music (Besson et al. 2007; Grassi et al. 2025; Herholz and Zatorre 2012; Kraus and Chandrasekaran 2010). Because this review asks how the brain and heart of a typical, untrained listener respond concurrently to music, studies of trained musicians were excluded, and we return to the implications of this choice in the Limitations.
4. Studies with a condition involving music listening, defined as listening that involves the cochlea. The lack of a universally accepted definition of music complicates its understanding across cultures and disciplines, particularly when equating auditory cognition with music cognition (Cross 2001; Nettl 2015). Given the existence of various definitions of music, we adopted a pragmatic approach, guided by the descriptions provided in the studies themselves, including only those studies that explicitly referred to or described the auditory stimulus as “music”.
5. The study must examine physiological responses during passive music listening, with or without accompanying psychological, emotional, or cognitive analyses. Studies involving music imagery, any state‐altering or concurrent manipulations before or during listening (e.g., exercise, pharmacological or mood induction, live performances, films) were excluded. Also excluded were studies on music's impact on physical performance, exercise recovery, or priming through language or imagery. No restrictions were placed on publication date or methodology if these criteria were met.
6. If any part of the study design met the inclusion criteria, that section was included, and data were extracted exclusively from those eligible segments. For instance, in O'Kelly et al. (2013), only the segment where healthy controls listened to recorded music met the inclusion criteria, and in Steinbeis et al. (2006) only the non‐musician data were of interest.

### 2.3 Information Sources

A systematic search of databases was conducted within PsycInfo (including PsycArticles), Pubmed, Scopus, Web of Science (core collection), and Academic Search Complete (EBSCOhost), using truncated/wildcard terms to capture all possible iterations. Search strategies were refined through consultations conducted with specialist library staff. Snowballing (citation searching) also revealed further relevant papers. The initial search was conducted on 31st January 2024. A final update search was conducted on 10th August 2025 to capture any studies published since the initial search. One additional paper was found and incorporated.

### 2.4 Search Strategy

The search query consisted of terms considered to describe the scoping review and its methodology: EEG, electroencephalography, ECG, cardiac, heart, music, listening. The search query was tailored to the specific requirements of each database. The search strategy for PsycInfo (including PsycArticles) is presented below. This search returned 117 results on 10th August 2025. Details of the complete search strategies for all databases can be found in Supporting Information—Appendix S2.

Any Field: eeg OR Any Field: electroenceph* OR Any Field: ERP OR Any Field: “evoked potential” OR Any Field: “neural oscillation” AND Any Field: ECG OR Any Field: cardi* OR Any Field: heart* AND Any Field: “music listening” OR Any Field: music* OR Any Field: listen* AND Population Group: Human AND Publication Type: Peer Reviewed Journal AND Peer‐Reviewed Journals only.

### 2.5 Study Selection

**FIGURE 1:**
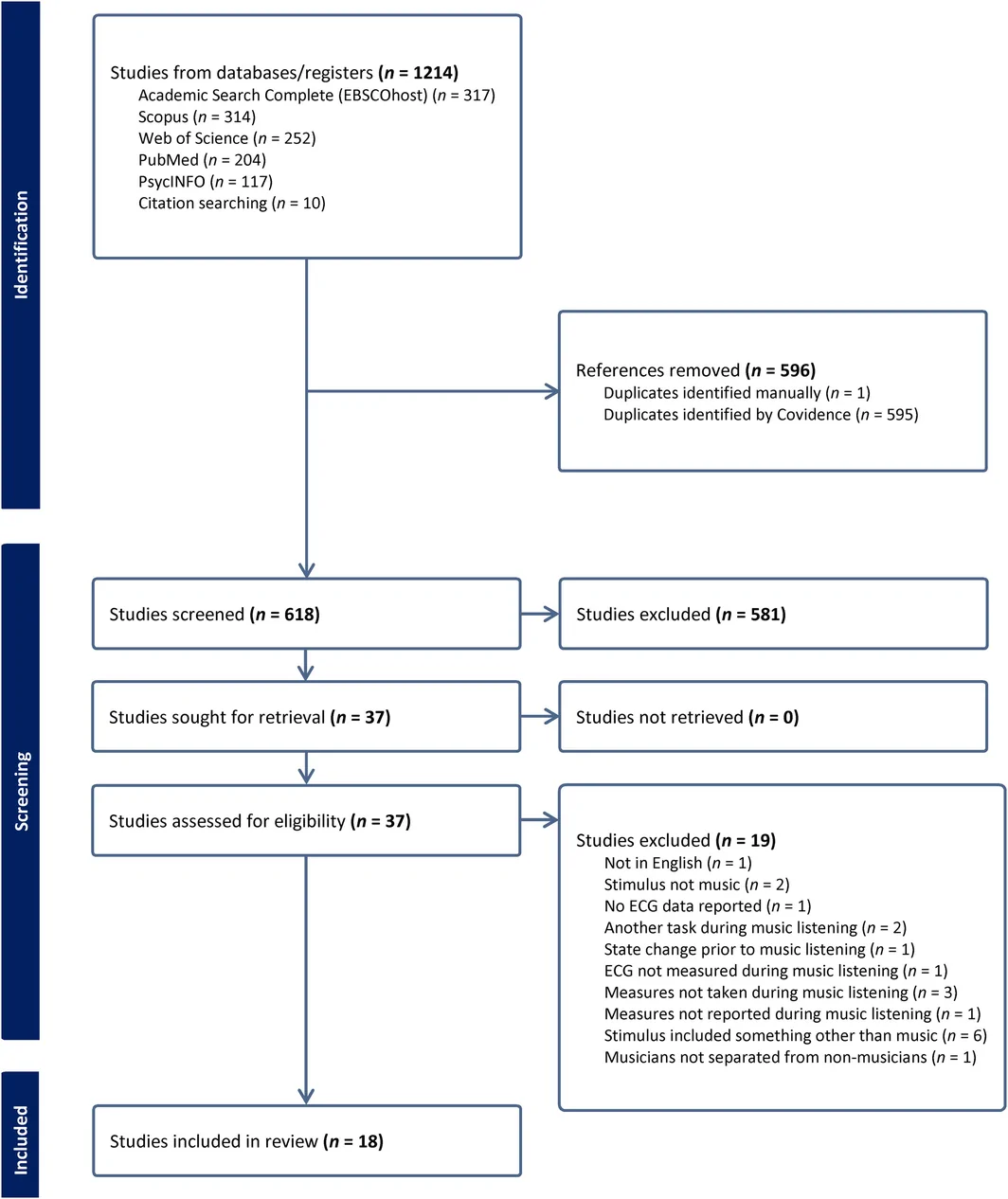
PRISMA flow diagram for the selection of studies.

Studies from the above database searches were transferred to the Covidence online software package (https://app.covidence.org/). Titles and abstracts of the studies uploaded were screened, and those not meeting the inclusion criteria were excluded, with the remaining being selected for full text review. A comprehensive analysis of the full text of those studies was conducted, with 18 meeting all criteria and being selected for data extraction. The PRISMA flow chart detailing this process is presented in Figure 1.

### 2.6 Data Charting Process

The Covidence data extraction template was iteratively tailored and refined during extraction to meet the goals and objectives of this review. The data were organized into six categories: publication information; participants (population); EEG and ECG measures (concept); and stimulus and study design (context). See Supporting Information—Appendix S3 for full details.

## 3 Results

Data were extracted from 18 studies that met the eligibility criteria for this scoping review as documented in the Supporting Information (see Appendices S2 and S3). The narrative that follows presents a breakdown of that information.

### 3.1 Publication Information

Table 1 shows the publication information extracted for each study with details below.

**TABLE 1:** Publication information.

Author/Year	Region	Aim of study	Findings	Brain‐heart interaction tested
Nanba et al. (1992)	East Asia	To investigate the effects of listening to modern music, categorized as stimulative or sedative, on EEG patterns, BP, HR, and Resp.	A transient decrease in right and left parietal Theta and Alpha was observed when both music types began; this activity then increased 2 min into the music. Theta increase to stimulative music was brief, to sedative music left maintained a high level throughout the music and right began to decrease after 3 mins. Alpha increased to stimulative but not sedative music. HR increased a little to stimulative music but not to sedative music. BP decreased during both stimulative and sedative music (both systolic and diastolic). Resp increased to stimulative but not sedative music.	No
Nanba et al. (1993)	East Asia	Investigate old Japanese music using EEG, BP, HR and Resp.	Left and right parietal Theta and Alpha showed significant increases during listening, with sex differences. BP and HR decreased but Resp increased slightly during music.	No
Baumgartner et al. (2006)	Western Europe	To examine the influence of visual and musical stimuli on brain processing using classical musical excerpts to evoke the three basic emotions of happiness, sadness and fear.	Alpha power density was largest (reflecting reduced brain activity) for sound conditions, intermediate for picture conditions, and lowest for the combined presentations. HR and Resp increased significantly during conditions involving music compared to visual only.	No
Steinbeis et al. (2006)	Western Europe	To explicitly test the hypothesis that harmonic expectancy violations are also emotionally significant.	Very unexpected chord (Neapolitan Sixth) elicited a distinct early negativity (EN) (~230 ms). The EN was followed by a slight positivity. Unexpected chord elicited an EN ~310 msec broadly distributed over the scalp, strongest over fronto‐central sites.^b^ No significant changes in the IBI for the three different types of harmonization in any of the three time windows (0–2 s, 2–4 s, 4–6 s).	No
Koelsch et al. (2008)	Western Europe	To investigate ERPs and HR in response to unexpected chords (as composed by classical composers) in a condition in which musical excerpts were played with musical expression by a pianist, and a condition in which excerpts were played without musical expression by a computer (no variation in tempo or loudness).	Both unexpected and highly unexpected chords triggered an early right anterior negativity (ERAN), whose amplitude nominally varied with harmonic irregularity (non‐significant). Emotional expression influenced N5 amplitude, without significant P3a effects, suggesting music syntax and emotion processing are distinct. No significant differences in IBI across chord types or expressiveness levels.	No
O'Kelly et al. (2013)	Western Europe	To investigate the effects of musical stimuli commonly used in music therapy with other auditory stimuli on neurophysiological and behavioral responses in individuals with disorders of consciousness.^c^	Liked music had greatest effect on right hemisphere EEG amplitude with similar increases to white noise in left. Distinct responses in frontal and temporal regions across bandwidths, with bias towards right hemisphere. Significant peak increase in Resp to liked music. Eyes Closed (EC) LF HRV approached significance, but change was non‐specific across stimuli. Significance for HR and HRV was less clearly defined in relation to effects of individual stimuli.^c^	No
Kuribayashi et al. (2014)	East Asia	To investigate the effects, and their time course, of inaudible high‐frequency components in high‐resolution music on EEG and HR.	High‐alpha EEG power significantly higher for full‐range music excerpts compared to high‐cut ones at left occipital region during last 50 s of listening, indicating a brain response to high‐frequency components without conscious awareness or changes in subjective feelings, heart rate, or facial expressions. No significant main or interaction effects of sound type for HR.	No
Jäncke et al. (2015)	Western Europe	To identify EEG oscillation time course patterns, and psychophysiological arousal, related to musical stimulus acoustic features and assess their stability during repeated exposure to the same piece of music.	Continuous increase in Event Related Synchronization in all frequency bands during music listening (relative to the pre‐music baseline). HR consistent across presentations. HR was lower in minute 2 of the 3‐min listening period. HR decreased with increased acoustic complexity and event‐related synchronization, despite an increase in EEG relative power and event‐related synchronization in central and parietal areas during listening.	Yes
Mollakazemi, Biswal, Evans, and Patwardhan (2018)	North America	To investigate the effects of tempo and cognition in music listening on eigen decomposition of cardiac‐synchronized EEG.	Eigen values decreased while listening to music, most significantly to slow song and particularly at P3. T4 showed the greatest change in EEG dimensionality: most concentrated variance during slow song, most distributed during favorite song. ECG measures were not reported.	Partial
Mollakazemi, Biswal, and Patwardhan (2018)	North America	To investigate the effects of tempo and cognition induced by auditory stimuli using cardiac‐synchronized EEG segments	Parietal Alpha most sensitive to auditory input. Gamma (right hemisphere) most sensitive to cognition. Favorite song caused largest changes in all EEG. Fast tempo song caused larger changes than slow. Alpha, Gamma and Gamma2 significantly affected by auditory input; Theta, least affected; Delta, no significant changes. ECG measures were not reported (Cognitive engagement was operationalized as a self‐selected favorite vs. an unfamiliar song).	Partial
Mollakazemi et al. (2019)	North America	To investigate the synchronization of autonomic and cerebral rhythms by music, specifically focusing on how tempo and familiarity of songs influence physiological responses.	Increase in coherence, indicating degree of synchronization, among cerebral, cardiovascular and respiratory rhythms during music listening. All songs significantly increased cerebro‐RR coherencies in the HF and LF band, slow tempo songs produced higher increases than fast tempo and larger effect sizes in frontal lobe and right hemisphere.	Yes
Teixeira Borges et al. (2019)	Western Europe	To investigate the relationship between scaling behavior in music and cortical dynamics in mediating music listening pleasure. Characterized the self‐similarity of fluctuations in loudness, pitch, and rhythm of 12 classical pieces and analyzed the scaling behavior of multiscale neuronal activity from different scalp regions and cardiac IBIs at baseline and during music listening, and associated these with self‐reported pleasure.	Implicated alpha, beta and gamma bands in music processing. Neuronal and cardiac dynamics showed a significant positive correlation during music listening. Music listening decreased the neuronal scaling exponent in temporal areas, linked to pleasure. Higher default‐state scaling exponents in pleased individuals were higher and approached those found in music loudness fluctuations. HR (AVNN) increased during music and was proportional to pitch scaling. Standard HRV measures were modulated by music, but α₁ (heart) was not consistently affected.	Yes
Mollakazemi et al. (2020)	North America	To measure changes in neuronal communication during music listening, using coherence, and to explore if any physiological variables including HR, BP, respiration and brain electrical responses became synchronized with songs.	Music listening increases synchronization among low‐frequency brain responses and physiological variables. Slow songs showed the highest, and fast songs the lowest, synchronization, with T4 showing the greatest synchronization, and the right hemisphere exhibiting larger average coherence compared to the left.	Yes
Kumarasinghe et al. (2021)^a^	Southeast Asia	To investigate the synchronization of the brain and heart responses to different auditory stimuli using complexity‐based analysis (fractal dimensions and sample entropy).	EEG complexity increases with music complexity, showing a strong correlation, while HRV complexity decreases in response to music, with both EEG and HRV complexity synchronizing to music's fractal dimension, indicating a unified response of brain and heart to musical complexity.	Yes
Mollakazemi et al. (2021)	North America	To investigate the effects of listening to music on EEG segments synchronized to different phases of the cardiac cycle, specifically focusing on how the tempo and cognition of music influence the complexity of brain responses in different brain regions.	Complexity of EEG responses increased with tempo and cognitive engagement, particularly for fast and favorite songs, with parietal and temporal lobes most affected; slow songs had a less complex, calming effect, indicating tempo and cognition have distinct impacts on brain activity, with cognition eliciting higher frequency responses and tempo affecting lower frequencies. ECG measures were not reported.	Partial
Namazi et al. (2021)^a^	Southeast Asia	To investigate coupling between EEG and ECG to changes in music complexity using SE of EEG and HRV.	Lower fractal complexity of the music stimulus led to greater change in EEG and HRV Shannon entropy. Music, EEG and HRV SE changes correlated. SE of EEG lowest at rest. Increased with each music presentation. Brown noise shows largest difference. EEG variations significant. HRV inverse of EEG: SE of HRV highest during rest and reduced with each music presentation. HRV variations not significant.	Yes
Sharma et al. (2021)	South Asia	To examine the anxiolytic effect of incremental modulations in tempo and octave.	Significant decrease in lower frequency EEG power in bilateral temporo‐parieto‐occipital regions compared to those who listened to SM or silence. SM group exhibited increase in higher frequencies, suggesting a different pattern of brain activity compared to the VM group. Significant decrease in HRV based on relative R‐R intervals for silence but not during music. No significant difference in other HRV parameters.	No
Kawashima et al. (2024)	East Asia	To investigate the brain‐heart interplay that is consistently observed across different mental states (music listening and rest).	A positive association between parieto‐occipital alpha2 power (10–12 Hz) in EEG and nHF, during both music listening and rest. This relationship persisted even after accounting for other variables such as mental fatigue, sleepiness, and emotional arousal, suggesting a robust brain‐heart interaction across different mental states.	Yes

*Note:* EEG “amplitude” denotes the magnitude of the EEG signal; “eigenvalues” summarize the dominant patterns of covariation within multichannel EEG; “scaling behavior” describes the long‐range temporal correlations (self‐similarity) of a signal (see Section 3.3.1). Where a source reported normalized HF power it is written nHF. The final column indicates whether the study quantified brain‐heart coupling (Yes), locked EEG to the cardiac cycle without quantifying coupling (Partial), or recorded both signals but analyzed them separately (No).

Abbreviations: BP, blood pressure; EEG, electroencephalography; ERP, event‐related potential; HF, high frequency; HR, heart rate; HRV, heart rate variability; IBI, interbeat interval (R‐R interval); LF, low frequency; Resp, respiration rate; SE, Shannon entropy; SM, stable music; VM, varying music.

^a^ Kumarasinghe et al. (2021) and Namazi et al. (2021) report the same dataset.

^b^ Only non‐musicians in this study met the inclusion criteria for reporting here.

^c^ Only healthy controls in the recorded music segment of this study met the inclusion criteria for reporting here.

#### 3.1.1 Author

There were 13 individual first authors of the 18 studies. Five of the 18 studies were by first author Mollakazemi (Mollakazemi, Biswal, Evans, and Patwardhan 2018; Mollakazemi, Biswal, and Patwardhan 2018; Mollakazemi et al. 2019, 2020, 2021) and two were by first author Nanba et al. (1992, 1993). Reports by Kumarasinghe et al. (2021) and Namazi et al. (2021) appear to be the same study.

#### 3.1.2 Year

The chronology of the studies shows that the majority (11) were published in the past 10 years, since 2015, with seven studies published prior to 2015. Of those seven, two were published more than 30 years ago, in 1992 and 1993.

#### 3.1.3 Region

Six studies were from Western Europe (including countries like the UK, Germany, France), five from North America, four from East Asia (China, Japan, South Korea), two from Southeast Asia (Indonesia, Malaysia), and one from South Asia (India, Pakistan).

#### 3.1.4 Aims and Findings

While all studies recorded and analyzed EEG and ECG, not all studies reported ECG, with three studies (Mollakazemi, Biswal, Evans, and Patwardhan 2018; Mollakazemi, Biswal, and Patwardhan 2018; Mollakazemi et al. 2021) using the ECG R‐peak solely as an internal timing marker to synchronize and extract short, cardiac‐locked EEG segments for examination.

### 3.2 Participants

Table 2 shows the participant information extracted from each study, with details of the data items extracted listed below.

**TABLE 2:** Participants.

Author/Year	Audiology	Musicianship	Handedness	Eligibility criteria	Sample size	Final number processed	Sex (Male:Female)	Age (years)
Nanba et al. (1992)			Claimed		35	18	9:9	University students
Nanba et al. (1993)			Claimed		50	32	16:16	18–25
Baumgartner et al. (2006)			Tested	Physical evaluation to screen out chronic diseases, mental disorders, medication, drug or alcohol abuse. Depression, anxiety and alexithymia were assessed with participants outside normal range excluded.	26	24	0:24	M = 26.1 SD = 5.3
Steinbeis et al. (2006)	Self‐report	No musical training beyond classroom music at school^a^	Claimed		12^a^	12^a^	6:6^a^	20–27^a^ M = 24.7
Koelsch et al. (2008)		No formal musical training besides normal school education	Tested		20	20	10:10	19–29 M = 24.7
O'Kelly et al. (2013)	Claimed by authors^b^	High level of musical proficiency excluded^b^			20^b^	EEG = 19 1 missing due to corrupted data^b^	7:13^b^	Male^b^ 29–59 M = 41 SD = 11 Female 24–52 M = 34 SD = 12.5
Kuribayashi et al. (2014)	Self‐report		Tested	Self‐reported normal hearing and vision and no history of neurological disorders	20	20	9:11	19–24 M = 21.2
Jäncke et al. (2015)	Examination	No history of musical training, as assessed by an in‐house questionnaire	Tested	No reported history of present or past neurological, psychiatric, or audiological disorders. Participants denied consuming illegal drugs or regular medication	16	16	4:12	Male M = 23.5 SD = 2.8 Female M = 22.2 SD = 8.7 Combined M = 22.53 SD = 7.47
Mollakazemi, Biswal, Evans, and Patwardhan (2018)	Examination				14	14	7:7	
Mollakazemi, Biswal, and Patwardhan (2018)					14	14		Adult subjects
Mollakazemi et al. (2019)	Examination			Exclusion: age < 18 years or > 37 years, pregnancy; hypertension; history of epilepsy or seizures; photosensitivity; adverse reaction after listening to certain music, songs or sounds; psychotropic drug use that could affect alertness or cognitive functions	14	14	7:7	18–37 M = 27.3 SD = 4.25
Teixeira Borges et al. (2019)		Were not musicians; only roughly half had any formal music training	Claimed	No history of neurological disease or psychiatric disorders	31	EEG = 28 ECG = 17	16:12	M = 26.8 SD = 4.2
Mollakazemi et al. (2020)					14	14	7:7	18–37
Kumarasinghe et al. (2021)				Did not drink alcohol/caffeine before the experiment	11	11	7:4	18–22
Mollakazemi et al. (2021)	Examination				14	14	7:7	18–37 M = 27.43
Namazi et al. (2021)				Had a “complete health condition” and did not drink alcohol/caffeine for 24 h before experiments	11	11	7:4	18–22
Sharma et al. (2021)	Claimed by authors	No history of music training	Claimed	Mental/neurological problems	25	21	21:0	18–24
Kawashima et al. (2024)					17	15	15:0	20–23

^a^ Non‐musicians only.

^b^ Healthy controls only.

#### 3.2.1 Audiology

Pre‐screening for hearing impairment, to ensure participants can perceive the stimulus, is essential but sometimes neglected. For most studies involving young typical participants, documenting self‐reported normal hearing is usually sufficient. However, the accuracy of self‐reports depends on the questions asked (Picton et al. 2000). Guidelines recommend that participants should be screened for normal hearing at 20 dB hearing level (dB HL) for the stimulus frequencies tested (Picton et al. 2000). Ten of the studies examined made no mention of hearing assessment. Of the eight that mentioned hearing assessment, four used an examination, two relied on participant self‐report, and two simply stated that participants had no history of, or known, hearing impairment.

#### 3.2.2 Musicianship

This review excluded studies which specified musicians as participants. A consensus in the music psychology literature is that a musician has at least 6 years of musical expertise (Zhang et al. 2020). Of the 18 papers examined, 12 made no mention of musicianship. Of the six papers that did mention assessment of musicianship, one used an in‐house questionnaire, one excluded those with a high level of proficiency, two mentioned participants having only a normal school education, one stated no history of music training, one stated that participants were not musicians and that only half had any formal music training. Notably, none of the studies employed standardized measures such as the Goldsmiths Musical Sophistication Index (Gold‐MSI) (Müllensiefen et al. 2014), which is widely used to assess individual differences in musical training and engagement.

#### 3.2.3 Handedness

Guidelines (Picton et al. 2000) suggest reporting of handedness for studies using motor responses, and handedness has been shown to be associated with auditory brain responses (Mohebbi et al. 2014), hemispheric asymmetries in the EEG spectrum (Ocklenburg et al. 2019), and cardiac measures (Yüksel et al. 2014). It would therefore seem pertinent to report handedness in studies examining EEG and ECG responses to music. Of the 18 studies examined, nine did not mention handedness. In five studies, handedness was claimed by the researchers without reference to assessment. Four studies assessed handedness, with three of those specifying the Edinburgh Handedness Inventory (Oldfield 1971), and one study (Baumgartner et al. 2006) simply stating it was tested “with standard handedness tests” (p. 35).

#### 3.2.4 Eligibility Criteria

Guidelines recommend reporting eligibility criteria, such as physical and mental health conditions, use of prescribed or illicit drugs, and consumption of substances like alcohol, nicotine, and caffeine, both during recruitment and on the day of recording, as these can affect both EEG and ECG measurements (Appelbaum et al. 2018; Jennings et al. 1981; Keil et al. 2014; Laborde et al. 2017; Picton et al. 2000; Quigley et al. 2024; Quintana et al. 2016; Quintana and Heathers 2014). Additionally, specific to cardiac measures on the day of recording, factors such as exertion, food and water intake, cardioreactive medications, drug abstinence, sleep patterns, and bladder fullness should be controlled (Jennings et al. 1981; Laborde et al. 2017; Quigley et al. 2024; Quintana et al. 2016; Quintana and Heathers 2014). The eligibility criteria mentioned above were missing in 10 papers, while eight papers reported various eligibility criteria as seen in Table 2.

#### 3.2.5 Sample Size

Guidelines (Appelbaum et al. 2018; Keil et al. 2022; Laborde et al. 2017; Picton et al. 2000) recommend that the sample size, or number of participants, be reported. All 18 papers adhered to this guideline, with participant numbers ranging from 11 to 50.

#### 3.2.6 Final Number Processed

Guidelines state that the achieved, or final, sample size should also be reported (Appelbaum et al. 2018). In seven papers, the final number of participants processed differed from the number recruited.

#### 3.2.7 Sex

Guidelines recommend reporting sex and age of participants (Appelbaum et al. 2018; Jennings et al. 1981; Keil et al. 2014; Laborde et al. 2017; Picton et al. 2000; Quintana and Heathers 2014). Only one study failed to report participants' sex distribution. In two studies, participants were exclusively male; one study (Baumgartner et al. 2006) had exclusively female participants, with a curious justification that “it is known that [females] show stronger emotional reactions than males” (p. 35). Sex was balanced in eight papers, with the ratio of male to female participants varying in the remaining six papers.

#### 3.2.8 Age

Three papers failed to report age details, with one of those providing no information, one stating participants were adults, and one stating participants were university students. The remaining 15 papers reported age statistics in various levels of detail: one paper noted only the mean age, while others provided more detailed statistics including both the age range and standard deviation.

### 3.3 Measures

#### 3.3.1 EEG Measures

Table 3 shows the EEG data extracted from each study. Papers were examined based on existing published guidelines for EEG measures (Keil et al. 2014; Picton et al. 2000; Pivik et al. 1993) which recommend details be reported regarding electrodes and their configuration, impedance, referencing, filtering, hardware and sampling frequency.

**TABLE 3:** EEG measures.

Author/Year	Variables	Electrode configuration	Electrode type	Impedance	References	Filtering	Hardware	Sampling frequency (Hz)
Nanba et al. (1992)	EEG spectral power measurements	10–20	Conventional				Nihon‐Kohden, SR 115‐S	
Nanba et al. (1993)	EEG spectral power measurements	10–20	Conventional				Signal processor (NEC‐San Ei, 7 T‐07A)	
Baumgartner et al. (2006)	EEG spectral power measurements	10–20	Ag/AgCl	< 5 kΩ	Recomputed to average reference	Digitally band passed to 1.5–30 Hz	Easy Cap System, Brain Vision amplifier, recorder and analyzer	500
Steinbeis et al. (2006)	Mean ERP values	10–20	Ag/AgCl		Left mastoid	Filtered off‐line using a band‐pass filter with a frequency range of 0.25–25 Hz (3001 points, FIR)	32/MREFA amplifiers	500
Koelsch et al. (2008)	Early right anterior negativity (ERAN) and the N5 ERPs	Extended 10–20	Ag/AgCl		Left mastoid as reference. Re‐referenced to the algebraic mean of the left and right mastoid.	0.25–25‐Hz band‐pass filter (1001 points, FIR)		500
O'Kelly et al. (2013)	EEG spectral power measurements	10–20			Common average montage	Hi/low cut off bandpass filter at 0.5 and 30 Hz.	XLTEC 50 channel video EEG and neuro‐physiological acquisition system	512
Kuribayashi et al. (2014)	EEG spectral power measurements	10–20			Average reference online; re‐referenced to the linked‐earlobe reference (A1, A2) offline.	Bandpass DC to 200 Hz	QuickAmp	1000
Jäncke et al. (2015)	EEG spectral power measurements	128‐Channel Geodesic		< 30 kΩ	Cz served as an on‐line reference	Band‐pass filtered at 0.1–100 Hz. band‐pass filtered (1–40 Hz) including a notch‐filter of 50 Hz.	128‐Channel Geodesic EEG system	500
Mollakazemi, Biswal, Evans, and Patwardhan (2018)	Amplitude eigenvalues 300 ms pre R peak	10–20					Biopac, IPS100	1000
Mollakazemi, Biswal, and Patwardhan (2018)	Eigen decomposition of the covariance of the data matrix of pre R peak EEG segments	Locations given				Band pass Butterworth IIR filter		1000
Mollakazemi et al. (2019)	LF and HF coherence of EEG with RR, SBP, DBP, and Respiration.	Locations given				The envelope function in MATLAB was used to compute the envelopes of the EEG. The envelopes, respiration and piecewise constant HR, SBP, and DBP were low‐pass filtered with a cutoff frequency of 5 Hz and sub‐sampled at a rate of 10 samples/s.	Biopac	1000
Teixeira Borges et al. (2019)	Scaling exponent of neuronal activity in various frequency bands	128‐Channel Geodesic	Ag/AgCl	< 50–100 kΩ range	Vertex	Non‐notch sinusoidal removal (PREP pipeline). Avoided standard high‐pass/notch filters to minimize long‐term signal distortion and preserve high‐frequency activity. Used a small sliding window (< 1 s) (Bigdely‐Shamlo et al. 2015)	128‐Channel Geodesic EEG system	1000
Mollakazemi et al. (2020)	MSC of low frequency time series extracted from envelope profile of EEG	Locations given				Envelope	Biopac	1000
Kumarasinghe et al. (2021)	Fractal dimension and sample entropy of EEG	10–20			A1 & A2	Removed DC offset then a fourth‐order Butterworth band‐pass filter (1–40 Hz)	Muse EEG	256
Mollakazemi et al. (2021)	Eigen decomposition of autocovariance of EEG segments synchronized to three different 300 ms segments of the cardiac cycle	Locations given				Bandpass Butterworth IIR filter. In total band, no bandpass filter was used, and 0.5–100 Hz and 0.5–125 Hz bands (filtered by the same bandpass Butterworth) were used to confirm the results of the total band.	Biopac	1000
Namazi et al. (2021)	Shannon entropy of EEG signals	Locations given			3 reference electrodes as shown in (Liu et al. 2020), fig.1	Removed DC offset then a fourth‐order Butterworth band‐pass filter (1–40 Hz)	Muse EEG	256
Sharma et al. (2021)	EEG spectral power measurements	10–10	Saline‐based				32‐channel EEG electrode cap (RapidCap) EEG‐ERP system	1024
Kawashima et al. (2024)	EEG spectral power measurements	10–20	Dry		Re‐referenced to all electrodes	Offline bandpass filter from 2 to 40 Hz	8 channel Neuroelectrics cap. StarStim (Neuroelectrics, Barcelona, Spain)	500

*Note:* Hardware details (manufacturer names, amplifier models, electrode counts) are reported as stated in the original publications. Where information is incomplete or inconsistent, this reflects the level of detail provided in the source study.

Abbreviations: Ag/AgCl, silver‐silver chloride; DBP, diastolic blood pressure; EEG, electroencephalography; ERP, event‐related potential; FIR, finite impulse response; HF, high frequency; IIR, infinite impulse response; LF, low frequency; MSC, Magnitude Squared Coherence; RR, *R*‐R interval (the IBI); SBP, systolic blood pressure.

To orient readers from other disciplines, we now briefly define the main classes of EEG measure encountered in the reviewed studies. One class of measure involves analysis of spectral power, which quantifies the amount of EEG activity within a given frequency band (e.g., alpha, 8–13 Hz). This measure is typically interpreted as an index of the engagement of the underlying cortical networks, with, for example, alpha power often increasing as a region becomes less actively engaged (González et al. 2021; Keil et al. 2022). A second class of EEG measure comprises event‐related potentials (ERPs), which are voltage deflections time‐locked to a specific event, such as the onset of an unexpected chord, and index the timing and magnitude of discrete processing stages (Picton et al. 2000). A third class of measures characterize the complexity or temporal structure of the ongoing EEG, rather than its power, at particular frequencies. Examples of this class of measures include (a) fractal dimension and entropy measures (e.g., sample entropy, Shannon entropy), which quantify how irregular or information‐rich the signal is, with higher values indicating more complex, less predictable activity (Kumarasinghe et al. 2021; Namazi et al. 2021); (b) the scaling exponent (or scaling behavior) which describes how the signal's fluctuations relate across time scales, indexing its long‐range temporal correlations or self‐similarity (Peng et al. 1995; Teixeira Borges et al. 2019); and (c) eigen‐decomposition‐based measures (eigenvalues), which summarize the dominant patterns of covariation within multichannel EEG, here applied to short segments locked to the cardiac cycle (Mollakazemi, Biswal, Evans, and Patwardhan 2018; Mollakazemi et al. 2021). The same measures can also be applied to the musical signal itself. Music is described as having a fractal structure because its fluctuations in loudness and pitch repeat a similar pattern at different time scales, so that a short excerpt is statistically self‐similar to a longer one (Levitin et al. 2012; Voss and Clarke 1975), and complexity studies ask whether the brain and heart follow that structure.

##### 3.3.1.1 Variables

Eight studies examined EEG spectral power. ERPs were examined in two studies. Advanced EEG signal analysis techniques such as amplitude eigenvalues, eigen decomposition, coherence measurements, fractal dimension, entropy calculations, and scaling exponent analysis were used in eight studies.

##### 3.3.1.2 Electrode Configuration

EEG guidelines (Keil et al. 2014; Picton et al. 2000; Pivik et al. 1993) state that standard electrode locations should be clearly defined whenever possible, with the standard electrode locations including the 10–20 system (Klem et al. 1999) and its revision to a 10–10 system proposed by the American Electroencephalographic Society (1994). Five studies detailed electrode locations but failed to mention the specific system used. The remaining 13 studies clearly stated the specific system used. The 10–20 system was most prominent, used in nine studies. One study described using the extended 10–20 system and one study used the 10–10 system. Two studies used EGI 128‐channel HydroCel Geodesic Sensor Nets (Geodesic Inc., Eugene, OR, USA). Guidelines by Picton et al. (2000) note that dense‐array systems, such as this, pose challenges in determining electrode placement as they surpass the 10–20 system's capacity and that, regardless of the naming convention employed, it is important to pinpoint landmark electrodes within the dense array that align with the standard sites in the 10–20 system. A technical note published by EGI describes how to determine the approximate correspondence between the international 10–10 electrode positions when using the 128 channel HydroCel Geodesic Sensor Net (Luu and Ferree 2005) however, the two papers using this system did not specifically mention if this process was conducted. While one of those papers (Jäncke et al. 2015) did use specific electrode naming conventions when discussing the EEG channels analyzed, the other (Teixeira Borges et al. 2019) did not mention specific electrode locations but used headmaps for data visualization.

##### 3.3.1.3 Electrode Type

Guidelines suggest that the EEG electrode type (passive or active), material (e.g., Ag/AgCl), and make and model, should be indicated (Keil et al. 2014; Picton et al. 2000; Pivik et al. 1993). Only eight studies mentioned electrode type, with four specifying Ag/AgCl electrodes, one study simply stating that the electrodes were saline‐based, one study mentioned dry electrodes, and two studies by the same author simply stated that ‘conventional’ EEG electrodes were used.

##### 3.3.1.4 Impedance

Picton et al. (2000) state that electrode impedances must be reported, while Keil et al. (2014) state that, although electrode‐scalp impedance is generally less important when using active electrodes, electrode impedance should be reported when appropriate. Only three studies reported impedance.

##### 3.3.1.5 Referencing

Referencing is an important methodological consideration because various reference locations provide distinct geometric perspectives for observing the underlying brain activity (Pivik et al. 1993) and guidelines state that referencing should be reported (Keil et al. 2014, 2022; Picton et al. 2000). Ten studies provided information regarding EEG referencing.

##### 3.3.1.6 Filtering

Guidelines state that while filters can be used to improve signal to noise ratio, they can lead to loss of information; therefore filtering must be recorded, including details of the type of filter, filter roll‐off, and cut‐off values (Keil et al. 2014, 2022; Picton et al. 2000; Pivik et al. 1993). Keil et al. (2014) note that simply stating the cut‐off frequency or frequencies is not enough; the filter family and/or algorithm, the filter order, and descriptive indices of the frequency response function should also be specified. Fourteen studies provided details regarding filtering.

##### 3.3.1.7 Hardware

Guidelines state that the make and model of the recording system should be indicated at a minimum (Keil et al. 2014) however, two studies omitted mention of the hardware used.

##### 3.3.1.8 Sampling Frequency

Guidelines state that sampling rate should be reported at a minimum (Keil et al. 2014) however, two studies by the same author made no mention of EEG sampling frequency. The remaining studies used various sampling frequencies ranging from 256 Hz to 1024 Hz.

#### 3.3.2 ECG Measures

Table 4 details ECG measures. Papers were examined based on existing published guidelines for ECG measures (Berntson et al. 1997; Jennings et al. 1981; Laborde et al. 2017; Quigley et al. 2024; Quintana et al. 2016; Quintana and Heathers 2014) which recommend details be recorded regarding the type of ECG measure (HR, HRV, other), electrodes and their configuration, recording equipment, filtering, sampling rate, and consideration of blood pressure and respiration effects on ECG.

**TABLE 4:** ECG measures.

Author/Year	Variables	Electrode configuration	Recording equipment	Filtering	Sampling rate (Hz)	BP/Resp
Nanba et al. (1992)	HR	Lead I	Takeda medical, TM 4010			Both
Nanba et al. (1993)	HR	Lead I	NEC‐San Ei, 7 T‐07A			Both
Baumgartner et al. (2006)	HR		PAR‐PORT			
Steinbeis et al. (2006)	R‐R time series (IBI)	Lead I	Two 32/MREFA amplifiers	2–60 Hz band‐pass filter (801 points, FIR)	500	
Koelsch et al. (2008)	R‐R time series (IBI)	Lead I				
O'Kelly et al. (2013)	HR; HRV LF	2 chest electrodes	XLTEC 50 channel video EEG and neuro‐physiological acquisition system			Resp
Kuribayashi et al. (2014)	HR	Lead II	QuickAmp		1000	
Jäncke et al. (2015)	HR	Lead I	Biopac MP100 amplifier		200	
Mollakazemi, Biswal, Evans, and Patwardhan (2018)	Cardiac synchronized EEG	Lead II	Spacelabs		1000	Both
Mollakazemi, Biswal, and Patwardhan (2018)	Cardiac synchronized EEG	Lead II			1000	
Mollakazemi et al. (2019)	HR; R‐R time series (IBI)	Lead II	Spacelabs	Low‐pass filtered with a cutoff frequency of 5 Hz and sub‐sampled at a rate of 10 samples/s.	1000	Both
Teixeira Borges et al. (2019)	HRV measures including time‐domain (AVNN, SDNN, RMSSD, pNN50), frequency‐domain (VLF, LF, HF, and LF/HF), and α₁ (heart)			A low‐pass filter (cut‐off frequency: 100 Hz) and a notch filter (~50 Hz) were applied during recording. After acquisition, high‐pass and low‐pass two‐way constrained least squares FIR filters (cutoff 45 Hz, order = 500; cutoff 0.5 Hz, order = 1200, −70 dB stopband attenuation, passband ripple = 0.1%) were used to eliminate baseline drift of non‐cardiac origin and minimize other artifacts such as power line interference or electromyographic noise.	1000	
Mollakazemi et al. (2020)	R‐R time series (IBI)	Lead II	Spacelabs	Low passed filtered by a cutoff frequency of 5 Hz and down‐sampled to rate of 10 samples/s.	1000	Both
Kumarasinghe et al. (2021)	R‐R time series (IBI)	5‐Lead ECG	Shimmer ECG		128	
Mollakazemi et al. (2021)	Cardiac synchronized EEG	Lead II	Spacelabs		1000	
Namazi et al. (2021)	R‐R time series (IBI)	5‐Lead ECG	Shimmer ECG		128	
Sharma et al. (2021)	HR, HRV LF/HF & rrHRV	Lead II on bilateral clavicles^a^				
Kawashima et al. (2024)	HRV LF & HF	Lead II	StarStim (Neuroelectrics)		500	

Abbreviations: α_1_, estimate of the short‐term scaling exponent of HRV between ~4 and 11 beats; AVNN, average of normal‐to‐normal intervals; BP, blood pressure; EEG, electroencephalography; FIR, finite impulse response; HF, high frequency; HR, heart rate; HRV, heart rate variability; IBI, interbeat interval; LF, low frequency; pNN50, percentage of normal‐to‐normal intervals; Resp, respiration rate; RMSSD, root mean square of successive differences; rrHRV, HRV based on relative R‐R intervals; SDNN, standard deviation of normal‐to‐normal intervals; VLF, very low frequency.

^a^ Not a standard Lead II placement.

As with the EEG, the cardiac indices reported fall into distinct classes that are not directly interchangeable. One class of measures involves heart rate (HR) and the interbeat interval (IBI; the time between successive R‐waves, also reported as the R‐R interval), which are time‐domain summaries of cardiac rate. Another class of measures includes heart‐rate variability (HRV), which quantifies the beat‐to‐beat variation in the IBI. This can be analyzed in the time domain (e.g., SDNN and RMSSD, which index overall and short‐term variability of the intervals, respectively) or the frequency domain (e.g., high‐frequency [HF] power, largely reflecting respiration‐linked parasympathetic influence, and low‐frequency [LF] power) (Nebe et al. 2023; Quigley et al. 2024). HF power falls in the frequency range of normal breathing, so it indexes the parasympathetic influence that reaches the heart with each breath and is sensitive to breathing rate and depth as well as to vagal tone (Berntson et al. 1997; Grossman and Taylor 2007), which matters here because music can change how a listener breathes (Bernardi et al. 2006). Time‐domain and frequency‐domain measures are computed from the same interval series but capture different aspects of it: time‐domain indices describe the size of the variability directly, whereas spectral indices describe how that variability is distributed across rhythms of different periods (Berntson et al. 1997). A third class of measures characterizes the complexity of the cardiac signal, using entropy or fractal measures of the IBI series to index the irregularity of the heart's dynamics rather than its rate or variance (Kumarasinghe et al. 2021; Namazi et al. 2021). These are computed from the same interval series as the HRV indices, but they describe the temporal structure of the fluctuations rather than their size, so we treat them as a separate class rather than a variant of HRV.

##### 3.3.2.1 Variables

Three studies used ECG to synchronize EEG measures to cardiac phase and did not report any cardiac measures. Cardiac measures examined include HR (eight studies), R‐R time series (Interbeat Interval; IBI) (six studies) and HRV (four studies). For HRV frequency domain measures, one study examined very low frequency (VLF) spectral power between 0.003 and 0.04 Hz, three studies examined low frequency (LF) spectral power between 0.04 and 0.15 Hz, two examined high frequency (HF) spectral power between 0.15 and 0.4 Hz, and two studies examined the ratio of low to high frequency power (LF/HF). It should be noted that current guidelines (Quigley et al. 2024) recommend not using LF or LF/HF ratio as measures of sympathetic activity; for a detailed discussion regarding issues with the LF/HF ratio measure see Billman (2013) and Heathers and Goodwin (2017).

Only two studies included time domain measures of HRV. One examined HRV time domain measures including: average of normal‐to‐normal (NN) intervals (AVNN); standard deviation of NN intervals (SDNN); square root of the mean of the squares of differences of successive heart periods (RMSSD); percentage of NN intervals > 50 ms (pNN50); and α₁ (heart), a unique index of short‐term HRV, calculated by estimating the short‐term scaling exponent of HRV between ~4 and 11 beats (Peng et al. 1995). The other study examined rrHRV, a measure of HRV based on relative R‐R intervals, using the difference of consecutive R‐R intervals weighted by their mean, introduced by Vollmer (2015). Current guidelines (Quigley et al. 2024) state that it is mandatory to conduct a comprehensive comparison and report when introducing a new HRV method, contrasting it against one or more established methods; however, the two studies that used unique measures, rrHRV and α₁ (heart), do not appear to have done so.

##### 3.3.2.2 Electrode Configuration

Precise electrode placement is essential for medical purposes but less critical for heart rate measurements used in psychophysiological research (Jennings et al. 1981). Guidelines recommend reporting of sensor placement, with Lead II, or modified Lead II, placement preferred (Jennings et al. 1981; Laborde et al. 2017; Quigley et al. 2024; Quintana et al. 2016). When using standard medical placements such as Lead II, it is recommended to specify both anatomical and medical designations following the 1975 American Heart Association guidelines (Pipberger et al. 1975). Two studies omitted details of electrode configuration. Various ECG electrode configurations were recorded, the most common being Lead II (eight studies). Of those studies, one (Sharma et al. 2021) reported “A single ECG channel (on bilateral clavicles; lead‐II)” (p. 117); however, this is not considered a standard, or modified, placement for Lead II, which is characterized by the voltage contrast between the left leg and the right arm, provided electrodes are situated beneath the inguinal fold anteriorly and the gluteal fold posteriorly for the former and below the shoulders for the latter (Kligfield et al. 2007; Pipberger et al. 1975; Quigley et al. 2024). Five studies used Lead I, and two studies used 5‐Lead electrode placement on the chest. One study simply stated using two chest electrodes. Finally, Jennings et al. (1981) mentioned that while electrode type and paste are minor factors in heart rate measurement, they still warrant mention, yet current guidelines omit these considerations.

##### 3.3.2.3 Recording Equipment

Guidelines recommend that details of recording equipment used (brand and model) be recorded (Jennings et al. 1981; Quigley et al. 2024; Quintana et al. 2016). Four of eighteen studies made no mention of the ECG recording equipment and, in the remaining studies, the details included were inconsistent, ranging from details of the specific ECG measurement device, name and brand used to simply mentioning the amplifiers used.

##### 3.3.2.4 Filtering

Guidelines state that details of filtering are important to enable methodological understanding and replication, and that real time or offline filtering of the raw signal should be clearly noted (Jennings et al. 1981; Quigley et al. 2024; Quintana et al. 2016; Quintana and Heathers 2014). Only four studies noted filtering.

##### 3.3.2.5 Sampling Rate

Guidelines (Berntson et al. 1997; Quigley et al. 2024; Quintana et al. 2016) recommend detailing sampling rate, with higher sampling rates reducing error in detecting the R‐wave. While lower sampling rates are feasible, 1000 Hz is the recommended frequency to reduce measurement error, particularly for HRV metrics (Quigley et al. 2024). Six studies made no mention of a specific ECG sampling rate. In those studies that did mention the sampling rate, the frequencies ranged from 128 to 1000 Hz, with 1000 Hz being most common (used in seven studies). While Berntson et al. (1997) in 1997 recommended 250 Hz as the lowest sampling rate for HRV, current guidelines (Quigley et al. 2024) state that sampling frequencies can go below 100 Hz for group comparisons provided the time series is interpolated prior to HRV calculation.

##### 3.3.2.6 Blood Pressure and Respiration

Guidelines (Jennings et al. 1981; Quigley et al. 2024; Quintana and Heathers 2014) recommend that data collection should include measures of respiration and vascular activity whenever possible, due to their influence on cardiac variables. Only five studies included both blood pressure and respiration measures, and one measured respiration only. All studies examined failed to mention controls for respiration, which can substantially affect the amplitude of HF HRV (Grossman and Taylor 2007; Quigley et al. 2024; Quintana and Heathers 2014). Only one of the studies that examined HRV included respiration as a measure (O'Kelly et al. 2013), however, the influence of respiration on HRV was not accounted for in that study.

#### 3.3.3 Synchronization, Peak Detection, and Cardiac Artifacts

Three issues become critical when EEG and ECG are recorded together. Each occurs with either signal alone, but here they act on the relationship itself. Poor alignment and imprecise R‐peak timing blur the temporal link, and cardiac contamination of the EEG can produce coupling where none exists. Reporting was sparse across the included studies.

##### 3.3.3.1 Synchronization

A first consideration is how the two physiological signals, and the musical stimulus, were aligned in time. Three arrangements are possible: recording EEG and ECG on a single integrated acquisition system, which guarantees sample‐accurate co‐registration of the two signals; recording them on separate devices synchronized by a shared trigger or clock; and aligning the physiological recording to the stimulus by means of an onset trigger. Where a single acquisition system was used, the two signals are inherently co‐registered even if the authors did not state this explicitly; this was the case, for example, in the studies using Biopac systems (Mollakazemi, Biswal, Evans, and Patwardhan 2018; Mollakazemi, Biswal, and Patwardhan 2018; Mollakazemi et al. 2019, 2020, 2021) and the integrated clinical acquisition system in O'Kelly et al. (2013). The studies that used the ECG R‐wave as an internal timing marker for cardiac‐locked EEG analysis (Mollakazemi, Biswal, Evans, and Patwardhan 2018; Mollakazemi, Biswal, and Patwardhan 2018; Mollakazemi et al. 2021) necessarily recorded both signals on the same clock, since the analysis depends on sample‐accurate alignment. In the remaining studies the recording arrangement, and in particular whether stimulus onset was captured by a hardware trigger or reconstructed afterwards, was generally not reported. Because any jitter between the stimulus, the EEG, and the ECG limits the resolution at which neural‐cardiac coupling can be measured, we recommend that future studies state explicitly how the two physiological channels and the stimulus were synchronized.

##### 3.3.3.2 Peak Detection

All heart‐rate and HRV measures derive from the detection of cardiac events. In every study reporting a cardiac measure, each heartbeat was detected from the R‐wave of the QRS complex, as is standard. However, the method of R‐wave detection, whether detection was automated or manually corrected, and how ectopic or misdetected beats were handled, was rarely described. This matters because errors in locating the R‐wave carry directly into the IBI series and hence into every derived HR and HRV metric, and because the short, cardiac‐locked EEG segments used by several of the included studies depend entirely on accurate R‐wave timing. We recommend that future studies report the peak‐detection procedure and any beat‐editing applied.

##### 3.3.3.3 Cardiac Contamination of the EEG

A further issue, which none of the included studies addressed, is contamination of the EEG by the electrical and mechanical activity of the heart. The cardiac field artifact (the heart's electrical signal spreading up to the scalp electrodes) and the ballistocardiographic or pulse artifact (movement of the electrodes with each pulse) both introduce cardiac‐locked activity into the EEG (Dirlich et al. 1997). This is a particular concern for the studies that analysed EEG segments locked to the cardiac cycle (Mollakazemi, Biswal, Evans, and Patwardhan 2018; Mollakazemi, Biswal, and Patwardhan 2018; Mollakazemi et al. 2021), because any residual cardiac field artifact is locked to the R‐wave and could be mistaken for, or inflate, a genuine neural response to the heartbeat; it is also relevant to any attempt to relate ongoing EEG power to cardiac activity. None of the reviewed studies reported steps to identify or remove cardiac artifact from the EEG. Methods for doing so are available: independent component analysis is the most widely used, and automated and regression‐based alternatives have since been developed and validated against it (Arnau et al. 2023; Dirlich et al. 1997; Tamburro et al. 2019). We note this as an important methodological gap and suggest that studies relating brain and cardiac signals should show that the neural effects they report cannot be explained by cardiac contamination of the EEG.

### 3.4 Experimental Conditions

#### 3.4.1 Stimulus

Table 5 shows stimulus details extracted from each study as discussed below.

**TABLE 5:** Stimulus.

Author/Year	Duration	Musical features	Style	Source	Delivery	Intensity	Selection	Familiarity	Attention
Nanba et al. (1992)	7 min	Composite	Dance and New Age	*Every Little Time* from Eurobeat and *Song for Peace* composed by Kitaro and played on a synthesizer.			Pre‐selected by investigator		
Nanba et al. (1993)	14 min 4 s	Composite	Japanese ceremonial music	Gagaku, an old Japanese ceremonial music form.			Pre‐selected by investigator		“by making them listen” (p. 95).
Baumgartner et al. (2006)	70 s	Composite	Classical orchestral pieces	Excerpts of exactly 70 s from: (1) Gustav Holst: *Mars the Bringer of War* from *The Planets*; (2) Samuel Barber, *Adagio for Strings*; and (3) Beethoven, *Symphony no. 6 (3rd mvt)*.			Pre‐selected by investigator		Instructed to place themselves into the same mood as expressed by the stimuli.
Steinbeis et al. (2006)	9–20 s	Harmony (cadence/expectation)	Classical chorales	Three matched versions of six Bach chorales, differing only by one chord to be harmonically expected, unexpected, and very unexpected. Taken from previously recorded MIDI files and altered in CuBase SX. Played without expressive features, tempo and loudness kept constant throughout. Score cited. Pieces and sections used provided in a table. Score example in figure.			Pre‐selected by investigator		Compared length of extract and indicated if longer or shorter than previous.
Koelsch et al. (2008)	8–16 s	Harmony (expectation)	Classical piano sonatas	25 excerpts from Sonatas by Beethoven, Haydn, Mozart, and Schubert, played by professional pianists and recorded to MIDI to create three harmonic versions of each excerpt (Expected, Unexpected, and Very Unexpected) by modifying a single chord, resulting in 75 unique files. For each of those 75 files a non‐expressive version was created by digitally removing all tempo and loudness variation, resulting in 150 stimuli in total. Score example in figure.			Pre‐selected by investigator		Button press to indicate detection of chords played with a deviant instrument.
O'Kelly et al. (2013)		Composite		Digital recordings of disliked music and white noise.		50–70 dB	Pre‐selected by investigator, participant selected from own collection		
Kuribayashi et al. (2014)	200 s	Inaudible HF components	Classical Baroque	First 200 s of *French Suite No. 5* by J. S. Bach played on cembalo (harpsichord), and a high‐cut version produced by digitally removing all frequencies above 20 kHz using a specialized low‐pass filter with an extremely steep slope (−1673 dB/octave). Supporting Information shows average power spectra of the two versions of the music.	Speakers	70 dB	Pre‐selected by investigator		Asked to listen to the music.
Jäncke et al. (2015)	Blocks 1 & 3, 18 min; Block 2, 15 min	Composite	Classical Opera	iTunes version of *Nessun dorma*, as sung by Paul Potts (~175 s duration).	HiFi earphones (Sennheiser, CX‐350)	75 dB	Pre‐selected by investigator	Familiar	Instructed to listen carefully to the music.
Mollakazemi, Biswal, Evans, and Patwardhan (2018)		Tempo		Slow & fast tempo songs, favorite song which “moved” them.	Circumaural headphones		Pre‐selected by investigator; participant selected from own collection	Both	
Mollakazemi, Biswal, and Patwardhan (2018)	3–4 min	Tempo		Slow & fast tempo songs, favorite song. Local phase randomized favorite and fast tempo songs.			Pre‐selected by investigator; participant selected from own collection	Both	
Mollakazemi et al. (2019)		Tempo		Favorite song. Fast and slow tempo vocal songs in Italian language. Favorite song and unknown (fast) songs processed for local phase randomization.	Circumaural headphones (mono)	A level that was comfortable	Pre‐selected by investigator; participant selected from own collection	Both	
Teixeira Borges et al. (2019)	111 s	Loudness, pitch & rhythm	Classical Piano compositions from the Humdrum Kern database	Two distinct compositions from six different composers obtained from Qobuz.com. Details of composer, performer and piece provided in Supporting Information the paper.	Speakers	65 dB	Pre‐selected by investigator	Both	Visual cue before each piece signaling to concentrate on the music.
Mollakazemi et al. (2020)		Tempo		Slow & fast tempo songs. Two songs, known and unknown, and two scrambled phase of local spectra versions of known and unknown songs.			Pre‐selected by investigator; participant selected from own collection	Both	
Kumarasinghe et al. (2021)	3 min	Complexity (embedded noise)	Classical	*Für Elise* by Beethoven, altered by white, pink & brown noise.^a^			Pre‐selected by investigator		
Mollakazemi et al. (2021)		Tempo		Slow and fast tempo songs. An unknown (fast) song and favorite song. Phase randomized version of known and unknown songs.	Circumaural headphones		Pre‐selected by investigator; participant selected from own collection	Both	
Namazi et al. (2021)	3 min	Complexity (embedded noise)	Classical	*Für Elise* by Beethoven, rhythm complexity altered by white, pink & brown noise.^a^			Pre‐selected by investigator		
Sharma et al. (2021)	8 min	Tempo & Pitch (octave)	Carnatic (Indian) classical music	Two custom recorded instrumental pieces played on Violin and Mridangam, one with incremental variations in tempo and octave. Details in text in Supporting Information of the paper.	Speakers	55–65 dB	Pre‐selected by investigator	Both	
Kawashima et al. (2024)	3 min	Composite		8 Pieces from the Music Emotion Recognition (MER) database plus 4 that were familiar and likely to evoke emotion.			Pre‐selected by investigator; participant selected from a limited set	Both	

Abbreviations: Both, familiar and unfamiliar; dB, decibels; HF, high frequency.

^a^ Also see (Hunt et al. 2014) for additional details.

##### 3.4.1.1 Duration

Various guidelines (Appelbaum et al. 2018; Keil et al. 2014; Picton et al. 2000; Quigley et al. 2024; Quintana et al. 2016) recommend reporting stimulus duration. Detailing stimulus duration in an experiment ensures consistency, reproducibility, and accurate data interpretation by controlling experimental variables. It also aids in understanding neurophysiological responses and behavioral implications, while ensuring methodological transparency. Five studies failed to detail stimulus duration. In the 13 studies that detailed stimulus duration, these ranged from 8 s to 18 min. These figures refer to the length of the musical stimulus. The total duration of the recording, including any baseline and post‐stimulus periods, was reported inconsistently across studies and could not be recovered for all of them, which is an example of the incomplete reporting discussed below.

##### 3.4.1.2 Musical Features

Music research frequently examines individual musical features such as rhythm, melody, and harmony, among others. Several studies investigated multiple distinct musical features (e.g., rhythm and pitch). These features were grouped into: composite (the music as a whole; 6 analyses); tempo (6 instances); rhythm (1); pitch (2); harmony (2); inaudible HF components (1); complexity (2); and loudness (1).

##### 3.4.1.3 Style

Considering the impact of even subtle variations in music style on musical taste and preference (Brisson and Bianchi 2020), and their ability to affect EEG and ECG measures (Merrill et al. 2023; Naser and Saha 2021), it is pertinent to consider musical style in this scoping review. Eleven studies reported this detail, with only one examining modern music (new age/dance). Ten studies involved classical music as stimuli, with eight using European classical music, one using Japanese classical music, and one using Indian classical music. Seven studies did not provide specific details of the stimulus musical style.

##### 3.4.1.4 Source

The details of the specific source of a piece of music are crucial in music research because variations in musical elements such as tempo, instrumentation, phrasing, and pitch can significantly alter the listening experience and its effects (Chilvers et al. 2024; Eerola et al. 2013; Juslin and Laukka 2004; Juslin and Västfjäll 2008). Simply stating the name of a piece of music is insufficient, as the same piece can be performed or recorded with a wide range of different musical elements. These variations can lead to different emotional and physiological responses, impacting the outcomes of any music listening research paradigm. Therefore, accurately detailing the specific version and characteristics of the music used is essential for ensuring the validity and reproducibility of research findings. This may seem obvious, but we are unaware of existing guidelines regarding this in the music research domain. The reporting of the music stimulus details varied greatly across the 18 studies, as seen in Table 5. Some studies simply named the general style and many simply named the composition, with some of those stating where it was sourced. Three studies specified segments used: one simply provided composition names and durations of excerpts; one provided both the composition names, sections, and specific bars used in a table, plus a score example in a figure; and a third provided names of pieces and a score example in a figure. Crucially, only two papers mentioned Supporting Information: one listed composer, performer, and piece details, and the other noted further descriptive details. None of the 18 studies directly provided the actual score for all stimuli, audio files, or links to such, representing a significant gap in methodological transparency. The lack of detail regarding the actual stimulus used makes direct replication of these studies impossible.

##### 3.4.1.5 Delivery

Guidelines recommend that the delivery method (e.g., headphones or speakers) should be specified because it can create a different listening experience (Keil et al. 2014; Robb et al. 2011). In this review, only seven studies provided this essential detail: three specified speakers, three used headphones, and one specified earphones. Only one study specified the brand and model of the equipment used to present the music. Eleven studies provided no details regarding how the music was presented. Surprisingly, only one study specified the number of channels (mono). It might be assumed the others were presented in stereo, but that detail was not reported.

##### 3.4.1.6 Intensity

Intensity, volume and loudness are interrelated concepts in the context of sound. Intensity refers to the physical power of a sound wave, measured in decibels (dB). “Volume” is often used interchangeably with “loudness”. Loudness is the subjective perception of the sound's strength, influenced by intensity, frequency, and other physical parameters such as spectral content, bandwidth, and temporal properties like duration (Schmidt et al. 2020). Higher intensity generally leads to higher perceived loudness (Olsen et al. 2015). In the context of music, being sound presented over time, intensity can be measured as either: (a) peak intensity, which captures the loudest moments in music; and/or (b) average intensity, reflecting the overall loudness of the music over time. Measuring the intensity, volume, or loudness of a sound stimulus is important not only for auditory health but also because the volume of auditory stimuli is a major determinant of changes in physiological measures and has been positively correlated with attention, the orienting response, and arousal (Coutinho and Cangelosi 2011; Gabrielsson and Lindström 2010; Huron 1992). Findings in psychophysical experiments may be affected by the methods used to judge loudness and intensity (Namba 1987). Changes in loudness in a musical context can result in changes in psychophysiological variables such as HR (Chuen et al. 2016) and EEG (Schmidt et al. 2020). Guideline recommendations for both EEG and ECG state that intensity needs to be considered for its impact on these measures and should be reported in dB, including the specification of whether this relates to sound pressure level (SPL), sensation level (SL), hearing level (HL), or another operational definition (Keil et al. 2014; Koelsch and Jäncke 2015).

Only six of the 18 studies reported the intensity of the stimulus, and of these, one simply stated that the volume was at a level that was comfortable to the participant. In the five studies that detailed intensity, the volume levels ranged from 50 to 75 dB, and none mentioned if this was SPL, SL, or HL, or if the intensity reported was the peak or average intensity, something quite relevant for physiological responses to music stimuli.

##### 3.4.1.7 Selection

Reporting guidelines for music interventions (Robb et al. 2011) recommend that stimulus selection be reported using four categories: pre‐selected by investigator; participant selected from a limited set; participant selected from own collection; and tailored based on participant assessment. Methodological recommendations for cardiac studies involving music (Koelsch and Jäncke 2015) suggest that participants choose their stimulus from a selection prepared by the researcher. The latter approach is suggested to balance control over variables while accommodating individual preferences, enhancing the reliability and validity of research findings. All studies examined featured stimuli pre‐selected by the investigator, with six studies also involving participant selection from their own collection, and another study also involving participant selection from a limited set.

##### 3.4.1.8 Familiarity

Individual factors like preference, familiarity, cultural background, past experiences, and perception of musical structure can vary widely and greatly influence how people respond psychologically to music (Chilvers et al. 2024; Freitas et al. 2018; Sarasso et al. 2022; Schaefer 2017). Strength of music preference is related to its function, with one of the primary functions of music being its use in regulating emotions, mood, and physiological arousal (Schäfer 2016; Schäfer et al. 2013). Musical preference is known to be related to cognitive style (Greenberg et al. 2015) and impacts cognition (Caldwell and Riby 2007) and physiological response (Merrill et al. 2023). Familiarity increases music preference (Fuentes‐Sánchez et al. 2022) and impacts brain response (Freitas et al. 2018; Jagiello et al. 2019; Kumagai et al. 2017; Pereira et al. 2011), cardiac measures (Kirk et al. 2022), and coupling between cardiac and brain activity (Mishra et al. 2022). Furthermore, the origin of music stimuli and its mismatch with the participant's cultural or linguistic background alters familiarity and expectancy (Morrison and Demorest 2009; Stevens 2012). Therefore, it is important to assess aspects of familiarity when examining physiological responses to music listening.

Nine studies noted participant familiarity with the music, using stimuli that were both familiar and unfamiliar. However, the issue of stimulus familiarity was handled inconsistently: in several cases, familiarity was either assumed or changed during the study. For example, one study (Jäncke et al. 2015) selected the stimulus because it was considered well known but did not assess initial participant familiarity. This same stimulus, which was around 3 min in duration, was then played repeatedly across three blocks (totalling 51 min), as the study was specifically interested in responses to repeated presentation of the stimulus. Consequently, participants who were initially unfamiliar with the music would certainly have gained familiarity by the end of the session. In another (Mollakazemi et al. 2019), songs sung in Italian were chosen specifically because the participants did not speak Italian, yet familiarity was not assessed. Another study (Sharma et al. 2021) used custom recorded music, which was inherently unfamiliar, but involved repeated listening over 6 days, increasing familiarity over time.

##### 3.4.1.9 Attention

While physical features of auditory stimuli are processed whether they are attended or not (Näätänen 1990), auditory attention in music listening is critical because it shapes our perception by aligning with our goals and expectations, guiding our focus to specific elements, and enhancing our overall listening experience (Fritz et al. 2007). The same musical piece can evoke entirely different neural processing and physiological responses depending on how attention is directed to it (Jäncke et al. 2018; Loui et al. 2005). Guideline papers in general appear to overlook attention to stimulus. Only seven studies noted how the participants' attention was directed to the stimuli. Three did so using instructions to listen, with one (Nanba et al. 1993) simply stating “by making them listen” (p. 95), and two asked participants to listen or listen carefully, without further detail. One study presented a visual cue before the music to prompt concentration, and another instructed participants to adopt the mood of the music. Two studies measured active attention by requiring participants to respond to specific aspects of the music, such as extract duration or detection of deviant instruments.

#### 3.4.2 Study Design

Table 6 shows the study design data extracted from each study, details of which are below.

**TABLE 6:** Study design.

Author/Year	Hypothesis	Room details	Time of day	Orientation	Visual state	Control/Baseline	Power analysis	Ethics/Informed consent
Nanba et al. (1992)		Temperature controlled, air‐conditioned		Seated	Blindfolded	Before: 7 min (first 2 min excluded) rest; After: 5 min rest		
Nanba et al. (1993)		Temperature controlled, air‐conditioned		Seated	Eyes covered with eye bandages	Before: 7 min (first 2 min excluded) rest; After: 5 min rest.		
Baumgartner et al. (2006)	That the combined presentation of two emotional stimuli will result in increased activation in emotional brain structures and correspondingly increase subjective and psychophysiological arousal			Seated (with head in a chin rest)	EO—Fixation cross	Compared music, music + visual, visual (no neutral control)		Both
Steinbeis et al. (2006)	That increasing harmonic unexpectedness would heighten subjective emotion, perceived tension, physiological arousal, and elicit specific brain responses.^a^				EO	EEG: averages computed with a 2500 ms prestimulus baseline^c^, ECG: averages computed with a 1 s prestimulus baseline^c^		
Koelsch et al. (2008)	That unexpected harmonies, compared to expected, would increase both physiological arousal and specific neural responses. That musical expression would further increase physiological arousal but not influence brain responses.				EO—Fixation cross	EEG: 200‐ms prestimulus baseline for each ERP. ECG baseline not reported.^c^		Both
O'Kelly et al. (2013)					EO, EC; only EC analyzed.^b^ “Instructed to close their eyes half way into each stimulus presentation to provide both eyes open and closed data” (p. 4).	Before: 5 min silence		Ethics only
Kuribayashi et al. (2014)					EO—while looking at a landscape picture	Non‐hi‐cut music (control)		Both
Jäncke et al. (2015)		Sound‐shielded		Seated	EC	5 s baseline immediately before music presentation		Both
Mollakazemi, Biswal, Evans, and Patwardhan (2018)					EC	No song (control)		Ethics only
Mollakazemi, Biswal, and Patwardhan (2018)	That interactions between cardio‐cerebral rhythms cause changes in neuronal oscillations when auditory stimuli are synchronized with cardiac rhythm.			Seated	EC	10 min no song (control)		Both
Mollakazemi et al. (2019)		Normal indoor conditions (temperature, humidity)		Seated	EC	10 min, no music & no headphone over ears, before and after		Both
Teixeira Borges et al. (2019)	That the brain's sensitivity and pleasure from music arise from the shared dynamical patterns between the music's scaling and the brain's neuronal scaling behavior.				EC	3 min EC rest before		Both
Mollakazemi et al. (2020)				Seated	EC	10 min no song (control)		Both
Kumarasinghe et al. (2021)		Isolated from external disturbances		Seated		3 min rest before		Both
Mollakazemi et al. (2021)					EC	10 min EC no song before		Both
Namazi et al. (2021)						3 min rest before		Both
Sharma et al. (2021)	That music with incremental tempo and octave modulations would produce a greater anxiolytic (anxiety‐reducing) physiological and psychological response compared to music without these changes or silence.	Dim‐lit sound‐attenuated chamber	Morning, Noon and Evening sessions (3 pm—7 pm)	Seated	EC	EEG analysis: 3 min silent rest (baseline) before intervention; and during 8 min intervention (stable/variable music vs. silence [control]) ECG: ‘resting state’ activity on the first vs. last day of each 1‐week intervention (music, silence). Note: Intervention involved 6 sessions over 1 week. See paper for full timing details.	✓	Ethics only
Kawashima et al. (2024)					EC	3 min EC rest before & after		Both

Abbreviations: EC, eyes closed; EO, eyes open.

^a^ Only hypotheses for non‐musicians reported here.

^b^ For healthy controls only.

^c^ Denotes an ERP preprocessing baseline (i.e., a pre‐stimulus epoch used for waveform correction), not a resting baseline condition.

##### 3.4.2.1 Hypothesis

Guidelines (Keil et al. 2014; Picton et al. 2000) suggest hypotheses and predictions should be clearly stated. Hypotheses were explicitly stated in six papers; others either reported research questions or appear to have been exploratory in nature (without explicitly claiming this).

##### 3.4.2.2 Room Details

Laboratory conditions such as temperature, lighting, and noise can impact physiological measures and should be reported (Appelbaum et al. 2018; Quigley et al. 2024). Twelve studies failed to mention the room conditions under which the experiment was run. Of the six studies that did mention room conditions, the details provided varied. Only three studies mentioned control of external noise, something quite pertinent to music listening.

##### 3.4.2.3 Time of Day

Although rarely reported, time of day can impact measures of HRV (Massin et al. 2000; Quintana and Heathers 2014; van Eekelen et al. 2004). HRV guidelines papers suggest that time of day should be reported and remain consistent across participants to control for circadian variation in HRV response (Laborde et al. 2017; Quigley et al. 2024; Quintana et al. 2016; Quintana and Heathers 2014). Only one study adhered to this recommendation.

##### 3.4.2.4 Orientation

Orientation (body posture) of participants is an important variable to note as it can impact both EEG (Rice et al. 2013) and ECG measures (Driscoll et al. 1995; Perini and Veicsteinas 2003). Cardiovascular guidelines specifically note that body posture and movement should be reported and controlled for (Jennings et al. 1981; Laborde et al. 2017; Quigley et al. 2024; Quintana et al. 2016). Nine studies mentioned orientation, and in all of these, participants were seated.

##### 3.4.2.5 Visual State

Visual state has been shown to impact cognition (Xu et al. 2014), and cognition to music specifically (Chang et al. 2015), as well as EEG (Barry et al. 2007; Barry and De Blasio 2017) and cardiac measures (Chethan et al. 2017; Hori et al. 2005). Two studies failed to mention the visual state of the participants during the experiment. The eyes‐closed (EC) visual state was more commonly assessed than the eyes‐open (EO) state. Nine studies reported the sole use of an EC visual state, one study reported using both visual states but assessed only the EC data, and two studies used the equivalence of an EC visual state; that is, one study reported that participants were blindfolded, and another reported covering participants' eyes with eye bandages. Four studies examined only the EO condition; of these, two reported the use of a fixation cross to reduce participant eye movement (a common approach), and one mentioned looking at a landscape picture. Details of the picture and the reason for using this method, or consideration of confounds introduced by looking at a landscape picture (having greater visual complexity than a fixation cross), were not mentioned.

##### 3.4.2.6 Control/Baseline

Baseline conditions are essential to control for individual differences and confounding variables, ensuring that observed changes can be accurately attributed to the experimental manipulation (Jennings et al. 1992; Keil et al. 2022; Quigley et al. 2024; Quintana and Heathers 2014). Quintana and Heathers (2014) noted that a baseline is “the non‐task situation that best controls for the presence of task comparison” (p. 6), and that the appropriateness of comparing a task to a resting baseline will vary. Guideline papers state that baseline instructions and periods should be noted, with the period being of sufficient length, adequately isolated from the stimulus onset to exclude any stimulus‐evoked activity, and containing no condition‐related differences (Keil et al. 2014; Quintana et al. 2016). Of the 18 papers reviewed, 13 included a resting baseline before the experimental condition; five of these also included a resting period afterwards. Six papers compared the experimental condition with a separate control condition. One paper included all three approaches, with a baseline before and after, as well as a control condition.

##### 3.4.2.7 Power Analysis

Guidelines state that a priori power analysis or methods used to determine the precision of parameter estimates should be reported (Appelbaum et al. 2018). Only one paper (Sharma et al. 2021) mentioned an a priori power analysis. While that study determined a sample size of 30 was required, only 25 participants were recruited, with final data analysis reduced to 21 participants.

##### 3.4.2.8 Ethics/Informed Consent

Ethical considerations are integral to academic institutions and publishing, with established guidelines and committees to approve and monitor research protocols to protect the rights of animal subjects and human participants. Investigators must adhere to the directives of these oversight bodies. Although these considerations are often implicit and not always explicitly mentioned in guidelines publications, institutional agreements, ethical standards, and informed consent are essential for any research involving human participants and must be documented (Appelbaum et al. 2018; Keil et al. 2014; McCauley and Christiansen 2019; Picton et al. 2000). Twelve of the papers examined mentioned both ethics and acquiring informed consent from participants, and three papers mentioned only ethics approval. Three studies, published in 1992, 1993, and 2006, did not explicitly report ethics approval and/or participant consent information, perhaps typical of research from that time period.

### 3.5 Findings

Table 7 shows the reported findings extracted from each study, organized by EEG measures, ECG measures, and EEG‐ECG interactions, with details discussed below. Across the 18 studies, EEG findings were highly heterogeneous, spanning spectral power changes across traditional frequency bands, event‐related potentials, complexity measures, and scaling exponents, with limited overlap in analytical focus. Cardiac outcomes were similarly varied, with studies reporting increases, decreases, or no significant change in heart rate, heart rate variability, or interbeat interval depending on stimulus type and context. Three studies (Mollakazemi, Biswal, Evans, and Patwardhan 2018; Mollakazemi, Biswal, and Patwardhan 2018; Mollakazemi et al. 2021) did not formally assess ECG measures, instead using the ECG R‐peak solely as a timing marker for cardiac‐synchronized EEG analysis. Direct examination of brain‐heart coupling was limited to a subset of studies, with preliminary evidence of synchronization between neural and cardiac rhythms during music listening.

**TABLE 7:** Reported findings.

Author/Year	EEG measures	ECG measures	EEG‐ECG interactions during music listening
Nanba et al. (1992)	Transient ↓ right & left parietal theta and alpha at music onset; ↑ after 2 min. Stimulative music: ↑ Theta (brief); ↑ Alpha. Sedative music: ↑ Theta (left parietal); ≈ Alpha.	Stimulative music: slight ↑ HR; ↓ BP (both systolic and diastolic); ↑ Resp. Sedative music: ≈ HR; ↓ BP (both systolic and diastolic); ≈ Resp.	
Nanba et al. (1993)	↑ left & right parietal theta and alpha, with sex differences.	↓ BP & HR; slight ↑ Resp.	
Baumgartner et al. (2006)	↑ Alpha power density.	↑ HR & Resp.	
Steinbeis et al. (2006)^a^	Very unexpected chord (Neapolitan Sixth) elicited distinct EN (~230 ms) followed by slight positivity. Unexpected chord elicited EN ~310 ms broadly distributed over the scalp, strongest over fronto‐central sites.	≈ IBI.	
Koelsch et al. (2008)	Unexpected and highly unexpected chords triggered ERAN (amplitude ≈ varied with harmonic irregularity). Expression influenced N5 amplitude (↑ for expressive vs. unexpressive performance); ≈ P3a.	≈ IBI.	
O'Kelly et al. (2013)^b^	↑ frontal right alpha and beta (liked music). ↑ frontal‐midline theta for liked vs. disliked music.	↑ Peak Resp (liked music). ≈ HRV, although increase in eyes‐closed LF HRV was near‐significant (*p* = 0.054).	
Kuribayashi et al. (2014)	↑ High‐alpha power in left occipital region during last 50 s (full‐range vs. high‐cut music).	≈ HR.	
Jäncke et al. (2015)	↑ Theta, low alpha‐1, low alpha‐2, upper alpha, and beta power (i.e., continuous ↑ event‐related synchronization). Positive correlations between acoustic variables (acoustic envelope, acoustic complexity) and power synchronization in each band.	↓ HR in minute 2 (of 3 min listening). Negative correlation between acoustic complexity and HR.	Negative correlation between HR and the event‐related synchronization in each band across the time course of music listening.
Mollakazemi, Biswal, Evans, and Patwardhan (2018)	↓ Eigenvalues; slow song produced most significant decreases (T3, T4, P3). P3 most sensitive across all songs. T4 showed the greatest change in EEG dimensionality: most concentrated variance during slow song, most distributed during favorite song.	Nil^c^	
Mollakazemi, Biswal, and Patwardhan (2018)	Parietal alpha most sensitive to auditory input. Gamma (right hemisphere) most sensitive to cognition. Favorite song caused largest changes. Alpha, Gamma & Gamma2 significantly affected; Theta least affected; Delta no significant changes.	Nil^c^	
Mollakazemi et al. (2019)	↑ coherence of EEG with cardiovascular and respiratory rhythms (see EEG‐ECG).	HR and IBI reported as part of coherence analysis (see EEG‐ECG).	↑ coherence among cerebral, cardiovascular & respiratory rhythms during music listening. Slow tempo songs ↑ higher than fast; larger effect sizes in frontal region & right hemisphere.
Teixeira Borges et al. (2019)	Alpha, beta & gamma bands implicated in music processing. Neuronal scaling exponent ↓ in temporal areas, linked to pleasure.	↑ HR (AVNN), proportional to pitch scaling. HRV measures modulated but α₁ (heart) not consistently affected.	Positive correlation between neuronal & cardiac dynamics during music listening.
Mollakazemi et al. (2020)	↑ synchronization among low‐frequency brain responses and physiological variables (see EEG‐ECG).	IBI reported as part of coherence analysis (see EEG‐ECG).	↑ synchronization among low‐frequency brain responses & physiological variables. Slow songs highest, fast lowest. T4 greatest synchronization; right hemisphere>left.
Kumarasinghe et al. (2021)	EEG complexity ↑ with music complexity, showing strong correlation.	HRV complexity ↓ in response to music though not statistically significant.	Both EEG & HRV complexity synchronized to music's fractal dimension, indicating unified brain‐heart response.
Mollakazemi et al. (2021)	Complexity of EEG ↑ with tempo & cognitive engagement, particularly for fast & favorite songs. Parietal & temporal lobes most affected; slow songs less complex; right hemisphere>left.	Nil^c^	
Namazi et al. (2021)	SE of EEG lowest at rest; ↑ with each music presentation. Brown noise (lowest complexity) music showed largest difference from rest.	SE of HRV highest during rest; ↓ with each music presentation (inverse of EEG). HRV variations not significant.	Music, EEG & HRV SE changes correlated.
Sharma et al. (2021)	Significant ↓ lower frequency EEG power in bilateral temporo‐parieto‐occipital regions for VM. SM group ↑ in higher frequencies.	Significant ↓ rrHRV for silence but not during music. No significant difference in other HRV parameters.	
Kawashima et al. (2024)	Parieto‐occipital alpha2 power (10–12 Hz) assessed (see EEG‐ECG).	nHF assessed (see EEG‐ECG).	Positive partial correlation between parieto‐occipital alpha2 power & nHF during both music listening (accounting for arousal & valence) & rest (accounting for fatigue & sleepiness).

*Note:* The nature/direction of the findings in the music listening condition are reported relative to the comparison condition/s for each assessed measure; ↑ = measure was relatively higher/greater during music listening; ↓ = measure was relatively lower/reduced during music listening; ≈ = measure showed relatively no significant change during music listening.

Abbreviations: α_1_, estimate of the short‐term scaling exponent of HRV; AVNN, average of normal‐to‐normal intervals; BP, blood pressure; ECG, electrocardiography; EEG, electroencephalography; EN, early negativity; ERAN, early right anterior negativity; ERP, event‐related potential; HF, high frequency; HR, heart rate; HRV, heart rate variability; IBI, interbeat interval; LF, low frequency; nHF, normalized high frequency; pNN50, percentage of normal‐to‐normal intervals; Resp, respiration rate; RMSSD, root mean square of successive differences; rrHRV, HRV based on relative R‐R intervals; SDNN, standard deviation of normal‐to‐normal intervals; SE, Shannon entropy; SM, stable music; VLF, very low frequency; VM, varying music.

^a^ Non‐musicians only.

^b^ Healthy controls only.

^c^ ECG measures were not formally assessed; ECG R‐peaks were used solely as a timing marker for cardiac‐synchronized EEG analysis.

#### 3.5.1 EEG

##### 3.5.1.1 Spectral Power

Studies assessing spectral power showed mixed effects across frequency bands. Alpha power changes were most frequently reported, with both increases and decreases depending on stimulus type and cortical region (Baumgartner et al. 2006; Jäncke et al. 2015; Kuribayashi et al. 2014; Nanba et al. 1992, 1993; Sharma et al. 2021). Theta, beta, and gamma bands were also implicated, though findings varied across studies (Mollakazemi, Biswal, and Patwardhan 2018; Nanba et al. 1992, 1993; Sharma et al. 2021; Teixeira Borges et al. 2019). One study reported continuous increases in event‐related synchronization across all frequency bands during music listening (Jäncke et al. 2015). High‐alpha power was found to be significantly higher for full‐range music compared to high‐cut versions in the left occipital region, suggesting sensitivity to inaudible high‐frequency components (Kuribayashi et al. 2014). Lower frequency EEG power decreased significantly in bilateral temporo‐parieto‐occipital regions in response to music with incremental tempo and octave modulations (Sharma et al. 2021).

##### 3.5.1.2 ERPs

Only two of the 18 studies assessed ERP components. Steinbeis et al. (2006) reported that unexpected chords elicited an early negativity (EN), with amplitude varying as a function of harmonic irregularity. Koelsch et al. (2008) reported that unexpected and highly unexpected chords triggered an Early Right Anterior Negativity (ERAN), and that musical expression modulated N5 amplitude, with greater N5 responses observed for expressively performed excerpts compared to unexpressive versions, while no significant P3a effects were found.

##### 3.5.1.3 Complexity

EEG complexity increased with music complexity, tempo, and stimulus familiarity (operationalized as listening to a self‐selected favorite versus an unfamiliar song), with parietal and temporal regions most affected (Kumarasinghe et al. 2021; Mollakazemi et al. 2021; Namazi et al. 2021). Slow tempo songs produced less complex EEG responses, consistent with a calming effect (Mollakazemi et al. 2021). Fractal dimension, sample entropy, and Shannon entropy analyses indicated that EEG complexity synchronized with the complexity of the musical stimulus (Kumarasinghe et al. 2021; Namazi et al. 2021).

#### 3.5.2 ECG

##### 3.5.2.1 HR

Heart rate findings were mixed across studies. Some reported modest increases to stimulative music (Nanba et al. 1992), others reported decreases (Nanba et al. 1993), and one found no significant main or interaction effect of sound type on HR (Kuribayashi et al. 2014). One study observed lower HR during the second of 3 min of listening, while also finding an inverse association between HR and acoustic complexity (Jäncke et al. 2015). HR and respiration rate increased significantly during conditions involving music compared to visual‐only conditions in one study (Baumgartner et al. 2006).

##### 3.5.2.2 HRV/IBI

No significant changes in interbeat interval (IBI) were found in response to harmonic expectancy violations in two studies (Koelsch et al. 2008; Steinbeis et al. 2006). HRV complexity, as measured by fractal dimension, sample entropy, and Shannon entropy, decreased in response to music, though these changes were not statistically significant (Kumarasinghe et al. 2021; Namazi et al. 2021). One study found a significant decrease in HRV based on relative R‐R intervals (rrHRV; a robust geometric HRV metric computed from the difference of consecutive R‐R intervals weighted by their mean, less sensitive to ectopic beats than absolute interval differences) for silence but not during music listening (Sharma et al. 2021). A caution applies to the absence of interbeat‐interval effects to harmonic expectancy violations noted above (Koelsch et al. 2008; Steinbeis et al. 2006). In both studies the violations fell at the close of a musical phrase or motive, so this null result may reflect the timing and placement of the manipulation rather than a general insensitivity of cardiac measures to harmonic expectancy.

#### 3.5.3 EEG‐ECG Interaction

Direct examination of brain‐heart coupling during music listening was limited to a small subset of the included studies. Where it was assessed, increased coherence among cerebral, cardiovascular, and respiratory rhythms was observed during music listening, with slow tempo producing higher synchronization and larger effect sizes in frontal and right hemisphere regions (Mollakazemi et al. 2019, 2020). Neuronal and cardiac scaling dynamics were positively correlated during listening (Teixeira Borges et al. 2019), and EEG and HRV complexity synchronized to the fractal dimension of the music (Kumarasinghe et al. 2021; Namazi et al. 2021). Heart rate was inversely related to event‐related synchronization in each frequency band across the time course of music listening (Jäncke et al. 2015). A robust positive association between parieto‐occipital alpha2 power and normalized high‐frequency HRV (nHF) persisted across both listening and rest conditions and was interpreted as suggesting a robust brain‐heart interaction across mental states (Kawashima et al. 2024). Taken together, the studies that analyzed both signals point the same way, towards closer coupling of neural and cardiac activity during music listening than at rest, but they do so using different measures, at different time scales, and in different frequency bands, so the agreement is one of direction rather than of detail. However, the small number of studies and the diversity of analytic approaches (coherence analysis, fractal dimension, scaling exponents) preclude firm conclusions.

## 4 Discussion

### 4.1 Publication Information

The fact that this scoping review identified only 18 papers for inclusion, with just 11 (61%) published since 2015, underscores a clear lack of recent literature assessing the effects of music listening in adult non‐musician populations using concurrent EEG and ECG measures. While numerous studies have examined EEG or ECG responses to music independently, very few have integrated both measures within the same experimental framework, limiting understanding of how neural and autonomic processes covary during music listening. Most studies reviewed (11 of 18; 61%) originated from Western Europe and North America. However, seven were conducted outside typical WEIRD contexts (Henrich et al. 2010), representing a relatively higher degree of global diversity than is commonly observed in music‐psychology research (Jakubowski et al. 2025). This suggests gradual progress towards broader inclusivity in sampling and cultural representation. Even so, cross‐cultural and intra‐cultural differences in how music is defined, perceived, and experienced remain substantial, reinforcing the need for continued diversification in both participant recruitment and stimulus selection. Broader cultural representation is essential to developing more inclusive and generalizable theories of music perception and response, as emphasized in recent calls to widen the scope of behavioral science beyond WEIRD populations (Apicella et al. 2020; Jakubowski et al. 2025).

### 4.2 Methodological Considerations

#### 4.2.1 Participants

Several methodological gaps were identified regarding participant details. In most studies (10 of 18; 56%) hearing assessment was overlooked. Assuming participants can perceive the stimulus without verification undermines the validity of a study, as it fails to ensure that all participants are equally experiencing the intended auditory stimuli, potentially skewing the results. Verification is essential to confirm that the measured effects are genuinely due to the stimulus and not influenced by unaccounted sensory limitations. Most studies (12 of 18; 67%) did not assess musical ability. Assessing musical ability is necessary in psychophysiological studies involving music listening because individuals with different levels of musical training process music differently, influencing cognitive and physiological responses (Dawson 2011). Nine (50%) of the 18 studies examined did not mention handedness. Accounting for neurophysiological and physiological variations between left‐ and right‐handed individuals is important when assessing EEG and ECG measures because it can affect brain lateralization (Ocklenburg et al. 2019) and cardiac autonomic function (Yüksel et al. 2014), potentially influencing the results and interpretation of data. While only one study failed to report the sex distribution of participants, it is suggested that precision in research and results can be further improved by surveying participants' sex assigned at birth and their current gender identity to distinguish between sex and gender (Nebe et al. 2023). Ten studies (56%) overlooked exclusion criteria during recruitment or on the day of the study, such as alcohol or caffeine consumption, and other potential confounds mentioned in the guideline literature (Jennings et al. 1981; Keil et al. 2014; Laborde et al. 2017; Mohebbi et al. 2014; Ocklenburg et al. 2019; Picton et al. 2000; Quigley et al. 2024; Quintana et al. 2016; Quintana and Heathers 2014; Yüksel et al. 2014). Among the studies that did address these factors, details varied greatly. These omissions highlight the need for more rigorous and standardized methodologies regarding participant screening in future research.

#### 4.2.2 Measures

It was found that many studies lacked reporting on critical aspects of EEG and cardiac measures, despite clear guidelines in the literature (Jennings et al. 1981; Keil et al. 2014; Malik et al. 1996; Picton et al. 2000; Pivik et al. 1993; Quigley et al. 2024; Quintana et al. 2016).

Focusing on EEG measures, 15 studies (83%) failed to mention impedance levels and 10 studies (56%) did not specify the type of electrodes used. Additionally, eight studies (44%) failed to mention the reference used, and four studies (22%) omitted filter details. Electrode type and impedance details are essential for assessing signal quality and reliability, while referencing methods are necessary to interpret EEG signals accurately in relation to a stable reference point, and filtering information is vital for understanding how the data were processed to extract meaningful brain activity patterns (Picton et al. 2000). Transparent reporting of these elements is essential for the reproducibility and robustness of EEG research and should not be overlooked.

Similarly, for cardiac measures, 14 studies (78%) did not report filtering details, 13 (72%) failed to record blood pressure, 12 (67%) did not include respiration (measurement or analysis), and six studies (33%) failed to report the sampling rate used. The omission of these details can compromise data validity. Specifically, transparent reporting of filtering is essential for the accurate interpretation and replication of both time and frequency domain analysis, as it mitigates noise sources like respiration and muscle artifact that directly distort the R‐peak location. This distortion corrupts the measurement of the IBI, the foundational measurement for HR and all HRV metrics and must therefore be reported (Quigley et al. 2024; Quintana et al. 2016). Furthermore, the simultaneous measurement of respiration is vital, as the high‐frequency component of HRV is physiologically linked to respiratory sinus arrhythmia. While indirect measures of respiration can be calculated from the cardiac intervals, the reporting of direct respiration measures should be considered as it provides necessary details like respiratory depth and a high‐fidelity signal for precise temporal assessment, which are essential for robust cardiovascular‐brain interaction studies (Zaccaro et al. 2018).

#### 4.2.3 Stimulus

Key aspects of stimulus description were lacking in the reviewed studies. Accurately characterizing both musical and acoustic features is essential for accurate analysis, interpretation, and replication of results. This includes core considerations such as musical genre, tempo, instrumentation, duration, and other objective stimulus details. While guidelines on music and auditory stimuli vary, they universally emphasize the need for clear reporting of these stimulus details (Koelsch and Jäncke 2015; Robb et al. 2011). However, numerous omissions were observed across key stimulus reporting domains. The common use of historical or formalized Classical music styles (employed in 10 studies; 56%) may be questioned in relation to contemporary participant familiarity and preference, suggesting a lack of ecological validity in stimulus selection, although classical music may offer practical advantages such as reduced participant familiarity effects and fewer copyright restrictions. It is noteworthy that none of the studies examined specifically mentioned using pop music, despite its ubiquity. As most papers provided limited detail about the musical material, we recommend improving transparency by providing sufficient identifying metadata (composer, performer, recording label, catalog number, year, and precise track timings) to allow independent retrieval of the stimulus. Where copyright permits, sharing sound files or detailed stimulus descriptions remains desirable. Long‐term and accessible storage solutions are important so that any material that can be shared remains usable over time.

This lack of comprehensive reporting is also pronounced regarding the delivery method. Eleven studies (61%) provided no information on whether music was delivered through speakers, headphones, earphones, or other means. Furthermore, only one of the 18 studies (6%) specified the brand and model number of the equipment used for presentation of the musical stimulus, and another (6%) mentioned whether the stimulus was presented in mono or stereo. Surprisingly, just five studies (28%) reported intensity in decibels. We recommend that future studies provide this crucial information about the delivery method to ensure replication and maximize data reliability. This includes reporting the make/model (at a minimum), headphone type (e.g., circumaural vs. supra‐aural vs. ear bud), or speaker configuration (e.g., position and number), and their frequency range/response (as equipment unsuitable for the stimulus frequency can distort results (Naal‐Ruiz et al. 2022; Soeta and Nishiura 2023)), and connectivity (wired vs. wireless). Comprehensive reporting of these equipment details is vital for maximizing the reliability and validity of music‐based interventions. However, not all omissions carry equal weight: fundamental parameters such as stimulus intensity in decibels represent more critical gaps than details such as mono versus stereo presentation, which, while desirable, are likely to have a smaller effect on outcomes.

A final, often overlooked issue relates to the implicit assumptions about how participants experience the stimulus. Nine studies (50%) mentioned familiarity with the music, and most studies (11 of 18; 61%) omitted reporting of participant attentional engagement with the stimulus. This lack of engagement reporting is significant, as participants in controlled laboratory settings may be prone to reduced attention, particularly when presented with unfamiliar or disliked musical styles (Kumagai et al. 2018). This issue may be further exacerbated if the individual does not perceive the stimulus as “music”, which is a critical assumption that researchers often fail to verify. Factors such as extreme manipulation, unfamiliarity, musical enculturation, or a lack of clear musical structure could lead participants to perceive stimuli as noise or an abstract sound (McDermott et al. 2016). We recommend that future studies explicitly include reporting of participant preference, familiarity, and attentional engagement with the stimulus.

#### 4.2.4 Study Design

Only six studies (33%) explicitly stated their hypotheses. Hypotheses are rarely emphasized in exploratory research to allow flexibility in exploring new ideas and discovering insights without predefined expectations. However, explicitly stating the research objective and intent, whether through a formal hypothesis or a declaration of exploratory goals, remains important. This clear framing guides the investigation, helps in evaluating findings, and allows for the precise testing of predictions. This practice enhances the study's credibility and reproducibility and is recommended in guidelines papers (Keil et al. 2014; Picton et al. 2000).

Room details such as lighting, temperature, and noise can significantly influence study outcomes by affecting participant comfort, cognitive performance, and physiological responses. However, despite guideline recommendations (Appelbaum et al. 2018; Quigley et al. 2024) these details were not mentioned in most (12; 67%) studies.

Time of day and posture (orientation) are important variables for consideration, particularly in relation to cardiac measures (Jennings et al. 1981; Laborde et al. 2017; Quigley et al. 2024; Quintana et al. 2016; Quintana and Heathers 2014) however, they were generally overlooked.

Regarding visual state, only one study (6%) examined both eyes open (EO) and eyes closed (EC), while 11 studies (61%) used only eyes closed conditions. As visual state has been shown to modulate emotional response and brain activity to music (Chang et al. 2015; Kallinen 2003), it would seem pertinent to consider the impact of these effects on study outcomes.

Statistical power is critical because it determines the likelihood of correctly identifying true effects in research studies. Higher power increases the chances of detecting smaller, yet potentially meaningful, effects, thereby enhancing the robustness and credibility of study conclusions. This is particularly important in ensuring that findings are not only statistically significant but also substantively meaningful in practical terms. A 2017 systematic review by Larson and Carbine (2017) found that none of 100 clinical EEG/ERP studies examined reported sample size calculations, and fewer than half included the statistics necessary for future power analyses. They recommended routine reporting of sample size justifications, effect sizes, and variance measures to improve reproducibility. In a 2019 analysis of 150 ERP studies, Clayson et al. (2019) found studies were typically aimed at detecting large effects, with an average sample size of 21. Similarly, a 2017 effect size distribution analysis of 297 HRV studies by Quintana (2017) revealed that HRV studies are generally underpowered due to small sample sizes. Clayson and Miller (2017) recommend conducting power analyses to determine the smallest detectable effect in a study context. Guidelines (Appelbaum et al. 2018; Clayson et al. 2019; Clayson and Miller 2017) stress using a priori sample size calculations to enhance methodological transparency and replicability. However, among the 18 studies reviewed, only one (6%) mentioned an a priori power analysis.

### 4.3 Findings

Overall, the studies indicate that music listening engages interconnected psychological and physiological mechanisms, reflected in concurrent changes in EEG and cardiac activity.

### 4.4 EEG

The diversity of EEG metrics employed, spanning spectral power, ERPs, complexity, and scaling exponents, combined with limited overlap in analytical focus, prevents directly comparable conclusions about the nature of EEG changes during music listening. However, several convergent patterns emerged: alpha power was the most frequently implicated band, complexity measures consistently increased with musical complexity and tempo, and parietal and temporal regions were most affected. That only two studies (Koelsch et al. 2008; Steinbeis et al. 2006) examined ERPs during music listening represents a notable gap, given the temporal precision ERPs offer for tracking moment‐to‐moment neural responses to musical events.

### 4.5 ECG

Different cardiac parameters capture distinct facets of autonomic regulation, which likely accounts for the mixed findings across HR, HRV, and IBI measures. The observation that HRV complexity decreased during music listening, albeit non‐significantly (Kumarasinghe et al. 2021; Namazi et al. 2021), while HRV based on relative R‐R intervals decreased during silence but not music (Sharma et al. 2021), suggests that music may exert a stabilizing influence on cardiac dynamics. However, the limited number of studies examining cardiac measures in detail, and the variation in stimuli, participants, and analytic methods, precludes firm conclusions.

### 4.6 Brain‐Heart Interaction

Direct examination of brain‐heart coupling was limited to a small subset of studies, yet where assessed, a consistent pattern of increased synchronization between neural and cardiac rhythms emerged during music listening (Kawashima et al. 2024; Kumarasinghe et al. 2021; Mollakazemi et al. 2019, 2020; Namazi et al. 2021; Teixeira Borges et al. 2019). A further study reported an inverse relationship, with heart rate decreasing as event‐related synchronization increased across the time course of listening (Jäncke et al. 2015). Slower tempi produced higher synchronization (Mollakazemi et al. 2019, 2020), and EEG and HRV complexity co‐varied with the fractal structure of the music (Kumarasinghe et al. 2021; Namazi et al. 2021). The finding that parieto‐occipital alpha2 power and nHF were positively associated across both listening and rest conditions (Kawashima et al. 2024) points to a brain‐heart interaction that may extend beyond task‐specific contexts. However, the diversity of analytic approaches and the small number of studies examining this interaction represent a significant gap, given the theoretical importance of brain‐heart coupling to understanding music's psychophysiological effects.

Although all included studies met the eligibility criteria, their heterogeneity precludes direct comparison of outcomes. Substantial variability was evident across aims, participant characteristics, stimuli, and analytic procedures. EEG and cardiac data were obtained using diverse hardware, electrode configurations, reference schemes, frequency bands, and outcome variables, alongside differences in stimulus type, duration, selection method, and baseline conditions. Such diversity, coupled with incomplete or inconsistent methodological detail, suggests that differences in findings likely reflect methodological and analytic variability as much as genuine physiological divergence. It is important to acknowledge, however, that some degree of heterogeneity may accurately reflect real individual differences in neural and autonomic responses to music. Accordingly, the collective evidence illustrates a growing but methodologically fragmented body of research, underscoring the need for greater standardization and transparency in future studies.

### 4.7 Limitations

Although this review was carried out transparently with pre‐registration on OSF and followed the standards set out in the PRISMA‐ScR (Tricco et al. 2018), it is important to acknowledge its limitations. Firstly, this review may have missed relevant literature despite our systematic attempt to be comprehensive. Secondly, while the data extracted were examined in relation to guidelines literature, not all aspects of the guidelines for EEG and cardiac measures were included due to the potential size and complexity this would entail. This scoping review was selective in the data extracted, focusing on what the authors considered most relevant. Finally, poor adherence to guidelines found in the papers examined may be due to the specificity of this scoping review as detailed in Section 2.2 and the relatively small number of papers examined (18), and may not be reflected in the broader literature. In addition, some omissions may reflect publication constraints, as authors are frequently limited by word counts and editorial decisions that may prioritize brevity over exhaustive description of methodological details.

A further limitation follows from our eligibility criteria. By design we excluded studies of trained musicians. While this removed a large and well‐documented source of variance and gave a cleaner reference point for the untrained listener, it also means the present review does not address how musical training shapes concurrent neural and cardiac responses to music, nor the differences and commonalities between trained and untrained listeners that are themselves informative for understanding the phenomenon. Extending this work to musician populations, ideally with musical expertise quantified on a standardized scale such as the Gold‐MSI (Müllensiefen et al. 2014), is an important direction for future reviews.

The review is also limited by the literature itself, as only a small number of the included studies analyzed the EEG and ECG data in relation to one another rather than as separate outcomes, so the conclusions this review can draw about brain‐heart interaction during music listening rest on a very small evidence base.

A related caution applies to the complexity measures. Fractal and 1/f analyses are often read as evidence of long‐range structure in music and in physiological signals, but that reading has been questioned (Colley and Dean 2019), so results from the studies using these measures are best treated as descriptive.

### 4.8 Summary and Conclusions

This scoping review provides the first systematic map of concurrent EEG and ECG use during passive music listening, synthesizing 18 peer‐reviewed studies across diverse methodological traditions. At the broadest level, the evidence is consistent with music having reliable psychophysiological effects. EEG activity and cardiac measures clearly respond to musical stimuli across studies and across diverse methodological contexts. The detail documented in Table 7 illustrates, however, that the direction and magnitude of these effects vary considerably: heart rate increases to some stimuli and decreases or remains unchanged to others; EEG effects span multiple frequency bands with limited overlap in analytical focus across studies. This heterogeneity is itself informative as it reflects not simply variability in individual responses, but a research field that has not yet coalesced around shared methodological standards. Methodological inconsistency across studies, as documented throughout this review, limits our ability to draw firm conclusions about the specific mechanisms through which music influences brain and heart activity. Clayson et al. (2019), in a study examining methodological reporting practices and statistical power in ERP research published from 2011 to 2017 across five prominent journals, found that failing to report key guidelines was ubiquitous, despite the publication of updated guidelines in 2014 aimed at enhancing methodological transparency. The present review corroborates this pattern in the context of combined EEG and cardiac music research, underscoring that methodological standardization is essential not only for scientific rigor but for enabling the kind of cross‐study synthesis that would allow firmer conclusions about music's psychophysiological effects.

A chronological pattern is apparent across the included studies, with earlier papers more likely to omit or under‐report methodological detail. This reflects the historical development of unified reporting guidelines and analytic techniques across psychophysiology and music science, not a failure of individual studies, but a field in the process of building shared infrastructure. Publication constraints and editorial word limits have also contributed to incomplete reporting across the period examined. The field has clearly progressed: the existence and increasing uptake of guidelines for EEG (Keil et al. 2014; Picton et al. 2000), cardiac measures (Quigley et al. 2024), and music‐based research (Koelsch and Jäncke 2015; Robb et al. 2011) provide a strong foundation for more rigorous future work, and the present review is intended as a contribution to that ongoing process.

The interdisciplinary nature of this research area presents both challenges and opportunities. Researchers from different backgrounds may lack clarity about what information is required for EEG, cardiac, or musical stimulus reporting, leading to under‐reporting of key methodological aspects. Within our own research team, comprising both music and psychophysiology specialists, collaboration proved invaluable. The process highlighted how concepts and terminology often taken for granted in one discipline can be unfamiliar or interpreted differently in another. These exchanges fostered mutual understanding and improved the rigor and inclusivity of the present review. More broadly, such interdisciplinary collaboration is essential for future progress, as it encourages shared methodological literacy, strengthens reporting standards, and promotes integrative interpretations of the neural and physiological mechanisms involved in listeners' responses to music. Moving forward, we recommend that future studies in this area: conduct a priori power analyses to determine appropriate sample sizes; screen and report participant characteristics consistently, including hearing acuity, handedness, musicianship, and day‐of‐recording exclusion criteria; provide comprehensive stimulus information including intensity in decibels, delivery equipment make and model, and systematic assessment of participant familiarity; report full EEG parameters including impedance, reference scheme, and filter specifications; include simultaneous measures of respiration when reporting HRV; and, where feasible, explicitly examine brain‐heart coupling rather than treating EEG and ECG as parallel but independent outcome measures. These steps would substantially improve comparability across studies and enable more meaningful synthesis in future reviews. Consistent reporting across both disciplines will reduce analytical variability, support reproducibility, and build the shared evidence base needed to advance understanding of the neural and physiological mechanisms underlying music's psychophysiological effects.

It is also important to be clear about what a scoping review of this kind can and cannot determine. The present review characterizes the methodological landscape of an interdisciplinary field as it currently stands; it maps existing practice against published guidelines rather than prescribing a single optimal experimental approach. Questions about the specific direction or magnitude of EEG or cardiac responses to particular musical features, or about the functional significance of brain‐heart coupling during music listening, ultimately require controlled experimental investigation with rigorous, standardized methodology. This review provides the field with a clear foundation for designing such studies. By identifying where current practice diverges from established guidelines, it defines the methodological baseline that future experimental work can build upon. The heterogeneity documented here is not a reason for pessimism about this research area, but is a precise characterization of what the next generation of studies needs to address. With further studies addressing these needs, we believe that the field will move from showing that music broadly influences EEG activity and cardiac measures to clearly delineating the ways in which music influences EEG activity and cardiac measures.

## Author Contributions

**Scott R. Leimroth:** conceptualization, methodology, investigation, data curation, formal analysis, visualization, writing – original draft, writing – review and editing, project administration. **Robert J. Barry:** conceptualization, methodology, supervision, writing – review and editing. **Frances M. De Blasio:** conceptualization, methodology, supervision, writing – review and editing. **Timothy P. Byron:** conceptualization, methodology, supervision, writing – review and editing.

## Funding

This research received no direct funding. S.R.L. was supported by the Australian Government Research Training Program.

## Disclosure

Acknowledgment of AI tool use: No generative AI tools were used in the preparation of this manuscript. Only standard grammar checkers and reference managers were used.

## Ethics Statement

The authors have nothing to report.

## Conflicts of Interest

Co‐author Robert J. Barry serves as Consulting Editor of *Psychophysiology*, Associate Editor of the *International Journal of Psychophysiology*, and Associate Editor of *Clinical Neurophysiology*. Co‐author Frances M. De Blasio serves on the Editorial Board of the *International Journal of Psychophysiology*. The authors request that these editorial roles be considered in editor assignment and reviewer selection. The other authors declare no conflicts of interest.

## Supporting information


**Appendix S1:** Framework (Population, Concept, Context).

**Appendix S2:** Search details.

**Appendix S3:** Data items.

## Data Availability

This scoping review did not generate or analyze primary experimental data; no original data or analysis code is associated with the manuscript. The review protocol was preregistered on the Open Science Framework at https://osf.io/yx3rh (associated project https://osf.io/xtznm). Full search strategies, eligibility criteria, and data‐charting items are reported in Appendices S1–, S3, and all data charted from the included studies are presented in the manuscript and Appendices S1–, S3.
