# Supplementary material for: Exploring EEG and ECG in Music Listening: A Scoping Review
Source: Psychophysiology. 2026 Sep 3;63(9):e70385. doi: 10.1111/psyp.70385 (PMC13542476; doi:10.1111/psyp.70385)
Supplement: Supplementary file 1 [file PSYP-63-e70385-s001.docx]

**Appendix S1**

**Framework (Population, Concept, Context)**

| Framework category | Inclusion criteria | Exclusion criteria |
| --- | --- | --- |
| Population | Conscious and awake, healthy adult (18+ yrs) human non-musicians | Animals; machine-learning models; clinical populations; children or adolescents; trained musicians. |
| Concept | Simultaneous EEG and ECG (or similar cardiac) recording during passive music listening, with both signals processed from the same session. ECG may serve as an internal timing marker for cardiac-locked EEG rather than as a cardiac outcome measure. | Studies that do not record EEG and ECG/cardiac activity simultaneously, or that record or process only one modality. |
| Context | Physiological responses to passive music listening are required; psychological, emotional, or cognitive outcomes may also be reported. | Any additional pre- or concurrent manipulation intended to alter the listener’s state or context beyond the music itself (e.g., stress or mood induction, pharmacological challenge, exercise, neurostimulation, visual or multisensory materials, guided imagery, meditation, live performance, or singing), or studies where the focus is not specifically on the music-listening experience. |
| Types of evidence/source | Peer reviewed published studies in English | Systematic reviews, meta-analyses, scoping reviews, narrative reviews, book chapters, conference and symposium abstracts, dissertation theses, pre-prints, protocols, and methodological-only studies. |
