# Supplementary material for: Exploring EEG and ECG in Music Listening: A Scoping Review
Source: Psychophysiology. 2026 Sep 3;63(9):e70385. doi: 10.1111/psyp.70385 (PMC13542476; doi:10.1111/psyp.70385)
Supplement: Supplementary file 2 [file PSYP-63-e70385-s002.docx]

**Appendix S2**

**Search Details**

| Source | Search terms |
| --- | --- |
| PsycInfo *(including PsycArticles)* | Any Field: eeg OR Any Field: electroenceph* OR Any Field: ERP OR Any Field: "evoked potential" OR Any Field: "neural oscillation" AND Any Field: ECG OR Any Field: cardi* OR Any Field: heart* AND Any Field: "music listening" OR Any Field: music* OR Any Field: listen* AND Population Group: Human AND Publication Type: Peer Reviewed Journal AND Peer-Reviewed Journals only |
| PubMed | All Fields ((((((((((EEG ) OR (electroencephalography)) OR (ERP)) OR ("evoked potential")) OR ("neural oscillation")) AND (ECG)) OR (cardiography)) OR (heart)) AND ("music listening")) AND (music)) AND (listen) Filters applied: Humans, English, Exclude preprints |
| Scopus | ( TITLE-ABS-KEY ( eeg OR electroenceph* OR erp OR "evoked potential" OR "neural oscillation" ) AND TITLE-ABS-KEY ( ecg OR cardi* OR heart* ) AND TITLE-ABS-KEY ( "music listening" OR music* OR listen* ) ) AND ( LIMIT-TO ( DOCTYPE , "ar" ) ) AND ( LIMIT-TO ( LANGUAGE , "English" ) ) AND ( LIMIT-TO ( EXACTKEYWORD , "Human" ) ) AND ( LIMIT-TO ( SRCTYPE , "j" ) ) |
| Web of Science *(core collection)* | ((ALL=(eeg OR electroencephalography OR ERP OR "evoked potential" OR "neural oscillation" )) AND ALL=(ECG OR cardiac OR heart)) AND ALL=("music listening" OR music OR listen)  English, Article |
| Academic Search Complete (EBSCOhost) | TX All Text, EEG AND ECG AND “music listening”, Boolean Phrase, Expanders: XApply related words, XAlso search within the full text of the articles, Limiters: XFull Text, XPeer Reviewed, XPublication, Type Academic Journal, XDocument Type: Article, XLanguage: English |
| Snowballing/Citation searching | Papers by Authors: Mollakazemi, Nanba |
