# Supplementary material for: Exploring EEG and ECG in Music Listening: A Scoping Review
Source: Psychophysiology. 2026 Sep 3;63(9):e70385. doi: 10.1111/psyp.70385 (PMC13542476; doi:10.1111/psyp.70385)
Supplement: Supplementary file 3 [file PSYP-63-e70385-s003.docx]

**Appendix S3**

**Data Items**

| Publication information | Author/Year Region (in which the study was conducted)   - North America - Western Europe (including countries like the UK, Germany, France) - Eastern Europe (including Russia, Poland, etc.) - East Asia (China, Japan, South Korea) - South Asia (India, Pakistan) - Southeast Asia (Indonesia, Malaysia) - Latin America - Oceania - Africa   Aim of Study  Findings |
| --- | --- |
| Population |  |
| Participants | Audiology Musicianship  Handedness  Eligibility Criteria  Sample Size Final Number Processed  Sex Age |
| Concept |  |
| Measures | EEG Measures   - Variables - Electrode Configuration - Electrode Type - Impedance - Referencing - Filtering - Hardware - Sampling Frequency   ECG Measures   - Variables - Electrode Configuration - Recording Equipment - Filtering - Sampling Rate - Blood Pressure/Respiration |
| Context |  |
| Experimental conditions | Stimulus   - Duration - Musical Component Examined - Style - Source - Delivery Method - Intensity - Selection - Familiarity - Attention to Stimulus |
|  | Study design   - Hypothesis - Room Details - Time of Day - Orientation - Visual State - Control/Baseline - Power Analysis - Ethics/Informed Consent |
